# Clinical laboratory test-wide association scan of polygenic scores identifies biomarkers of complex disease

**DOI:** 10.1186/s13073-020-00820-8

**Published:** 2021-01-13

**Authors:** Jessica K. Dennis, Julia M. Sealock, Peter Straub, Younga H. Lee, Donald Hucks, Ky’Era Actkins, Annika Faucon, Yen-Chen Anne Feng, Tian Ge, Slavina B. Goleva, Maria Niarchou, Kritika Singh, Theodore Morley, Jordan W. Smoller, Douglas M. Ruderfer, Jonathan D. Mosley, Guanhua Chen, Lea K. Davis

**Affiliations:** 1 Division of Genetic Medicine, Department of Medicine, Vanderbilt University Medical Center, Nashville, TN 37232 USA; 2 Vanderbilt Genetics Institute, Vanderbilt University Medical Center, Nashville, TN 37232 USA; 3 Department of Medical Genetics, University of British Columbia, Vancouver, BC V5Z 4H4 Canada; 4 Psychiatric & Neurodevelopmental Genetics Unit, Center for Genomic Medicine, Massachusetts General Hospital, Boston, MA 02114 USA; 5 Department of Psychiatry, Harvard Medical School, Boston, MA 02115 USA; 6 Stanley Center for Psychiatric Research, Broad Institute of Harvard and MIT, Cambridge, MA 02142 USA; 7 Department of Microbiology, Immunology, and Physiology, Meharry Medical College, Nashville, TN 37232 USA; 8 Analytic and Translational Genetics Unit, Center for Genomic Medicine, Massachusetts General Hospital, Boston, MA 02114 USA; 9 Department of Molecular Physiology and Biophysics, Vanderbilt University Medical Center, Nashville, TN 37232 USA; 10 Department of Psychiatry and Behavioral Sciences, Vanderbilt University Medical Center, Nashville, TN 37232 USA; 11 Departments of Medicine and Biomedical Informatics, Vanderbilt University Medical Center, Nashville, TN 37232 USA; 12 Department of Biostatistics and Medical Informatics, University of Wisconsin-Madison, Madison, WI 53706 USA; 13 Division of Genetic Medicine, Department of Medicine, Vanderbilt Genetics Institute, Vanderbilt University, 511-A Light Hall, 2215 Garland Ave, Nashville, TN 37232 USA

**Keywords:** Electronic health records, Population genetics, Genetic epidemiology, Biomarkers

## Abstract

**Background:**

Clinical laboratory (lab) tests are used in clinical practice to diagnose, treat, and monitor disease conditions. Test results are stored in electronic health records (EHRs), and a growing number of EHRs are linked to patient DNA, offering unprecedented opportunities to query relationships between genetic risk for complex disease and quantitative physiological measurements collected on large populations.

**Methods:**

A total of 3075 quantitative lab tests were extracted from Vanderbilt University Medical Center’s (VUMC) EHR system and cleaned for population-level analysis according to our QualityLab protocol. Lab values extracted from BioVU were compared with previous population studies using heritability and genetic correlation analyses. We then tested the hypothesis that polygenic risk scores for biomarkers and complex disease are associated with biomarkers of disease extracted from the EHR. In a proof of concept analyses, we focused on lipids and coronary artery disease (CAD). We cleaned lab traits extracted from the EHR performed lab-wide association scans (LabWAS) of the lipids and CAD polygenic risk scores across 315 heritable lab tests then replicated the pipeline and analyses in the Massachusetts General Brigham Biobank.

**Results:**

Heritability estimates of lipid values (after cleaning with QualityLab) were comparable to previous reports and polygenic scores for lipids were strongly associated with their referent lipid in a LabWAS. LabWAS of the polygenic score for CAD recapitulated canonical heart disease biomarker profiles including decreased HDL, increased pre-medication LDL, triglycerides, blood glucose, and glycated hemoglobin (HgbA1C) in European and African descent populations. Notably, many of these associations remained even after adjusting for the presence of cardiovascular disease and were replicated in the MGBB.

**Conclusions:**

Polygenic risk scores can be used to identify biomarkers of complex disease in large-scale EHR-based genomic analyses, providing new avenues for discovery of novel biomarkers and deeper understanding of disease trajectories in pre-symptomatic individuals. We present two methods and associated software, QualityLab and LabWAS, to clean and analyze EHR labs at scale and perform a Lab-Wide Association Scan.

**Supplementary Information:**

The online version contains supplementary material available at 10.1186/s13073-020-00820-8.

## Background

The overarching goal of this study was to determine whether laboratory (lab) test results collected in a hospital and outpatient setting could be mined against polygenic scores (PGS) to identify known and novel biomarker associations for complex disease. Lab test results are essential to routine clinical care. These targeted biochemical measurements facilitate disease diagnosis and influence health care delivery. Clinical lab values are also monitored as mediators of disease risk and are targeted by interventions to reduce disease incidence (e.g., cholesterol-lowering medication to reduce the risk of heart disease). Lab test results in the electronic health record (EHR) are a vast and growing resource for novel biomarker discovery, especially as EHRs are increasingly linked to patient DNA samples (e.g., the eMERGE consortium (https://emerge.mc.vanderbilt.edu)), the All of Us Program (https://allofus.nih.gov), and the Million Veteran’s Program (https://www.research.va.gov/mvp/)). Genetic studies of EHR-based labs could reveal novel biomarker-disease or biomarker-gene associations, which in turn could lead to better understanding of biological processes in disease, improved diagnostic algorithms, and new therapeutic targets.

Despite their potential, however, EHR-based labs have been used in only a handful of prior genetic studies [1–5], and none have systematically interrogated an extended collection of EHR-based lab values. Barriers to studying EHR-based labs include uneven data quality, and challenges inherent to analyzing and interpreting high-dimensional health care data. Data entry errors exist, resulting in implausible recorded values [6], some labs have different units and reference ranges over time, and many individuals have multiple observations of different lab tests, each measured at varying times relative to diagnoses and treatment [7]. Moreover, previous studies demonstrate that while 99% of lab results are accurately transmitted from the testing laboratory to the EHR, only 70% of test results contain all required reporting elements, and only 91% of results are appropriately formatted [8]. Thus, while these data represent real clinical care and may accelerate translational research, there is little precedent for their analysis and interpretation in genetic studies.

To address these challenges, we present a high-throughput framework for genetic analysis of EHR-derived lab data. We have developed two methods: the QualityLab pipeline to clean, standardize, and visualize lab data and the Lab-Wide Association Scan (LabWAS) pipeline to scan for associations between any variable of interest (genetic or otherwise) and the cleaned EHR labs. The LabWAS method is similar to the Phenome-Wide Association Scan (PheWAS) which scans for association between an exposure variable (typically, a genetic risk factor) and many phenotypes [9]. The PheWAS method has replicated many known gene-disease associations [10] and has identified novel pleiotropic genetic effects [11], opportunities for drug repurposing, and unintended drug consequences [12]. QualityLab builds off the success of previous measurement quality control methods, such as CLARITE [13]. While, CLARITE focuses on minimal cleaning of survey data, QualityLab conducts extensive cleaning of quantitative lab measurements derived from EHRs.

We hypothesized that EHR-based lab values could be used to identify known and novel relationships between genetics, biomarkers, and disease. We deployed our framework in the Vanderbilt University Medical Center (VUMC) EHR and linked biobank, BioVU, and replicated it in an independent biobank, Massachusetts General Brigham Biobank*.* We focused on genetic analysis of blood values of high-density lipoprotein cholesterol (HDL), low-density lipoprotein cholesterol (LDL), and triglycerides (TG) and on coronary artery disease (CAD) as proof-of-principle examples to test the association between PGS for CAD and known biomarkers of disease (LDL, HDL, and TG) using the QualityLab and LabWAS methods across populations. We show that EHR-derived lipids values are genetically similar to those in population-based studies and that PGS for lipids robustly associate with their respective lab in a LabWAS. Additionally, our LabWAS revealed that PGS for CAD associated with known lipid biomarkers, even in individuals without a history of CAD, and with potentially novel immune biomarkers.

## Methods

### Study sample

Our primary analysis was performed at VUMC which is a tertiary care center providing inpatient and outpatient care in Nashville, TN. The VUMC EHR was established in 1994 and includes data on billing codes from the International Classification of Diseases, 9th and 10th editions (ICD-9 and ICD-10), Current Procedural Terminology (CPT) codes, laboratory values, reports, and clinical documentation. The de-identified mirror of the EHR, known as the Synthetic Derivative, includes patient records on more than 2.8 million individuals. In 2007, VUMC launched a biobank, BioVU, and the BioVU Consent form is provided to patients in the outpatient clinic environments at VUMC. The form states policies on data sharing and privacy and, upon consent, makes any blood leftover from clinical care eligible for BioVU banking [14]. The VUMC Institutional Review Board oversees BioVU and approved this project.

### Genotyping and quality control

We obtained genotype information on 94,474 BioVU individuals of different ancestral and racial backgrounds genotyped on the Illumina MEGA^EX^ array. Using PLINK v1.9 [15], genotypes were filtered for SNP and individual call rates, sex discrepancies, and excessive heterozygosity (Additional file 1). We selected individuals of European or African ancestry using principal component analysis implemented in Eigenstrat [16, 17] and confirmed the absence of genotyping batch effects through logistic regression with “batch” as the phenotype. Imputation was completed using the Michigan Imputation Server [18] using the Haplotype Reference Consortium (HRC) reference panel. SNPs were then filtered for SNP imputation quality (*R*^2^ > 0.3) and converted to hard calls. We restricted to autosomal SNPs, filtered SNPs with minor allele frequency > 0.01, or with allele frequencies that differed by more than 10% from the 1000 Genomes Project phase 3 CEU or ASW set respectively [19], and Hardy-Weinberg Equilibrium (*p* > 1 × 10^−10^). The resulting dataset contained 6,303,629 SNPs on 72,824 individuals of European genetic ancestry and 12,798,111 SNPs on 15,283 individuals of African genetic ancestry.

### QualityLab pipeline

**Fig. 1:**
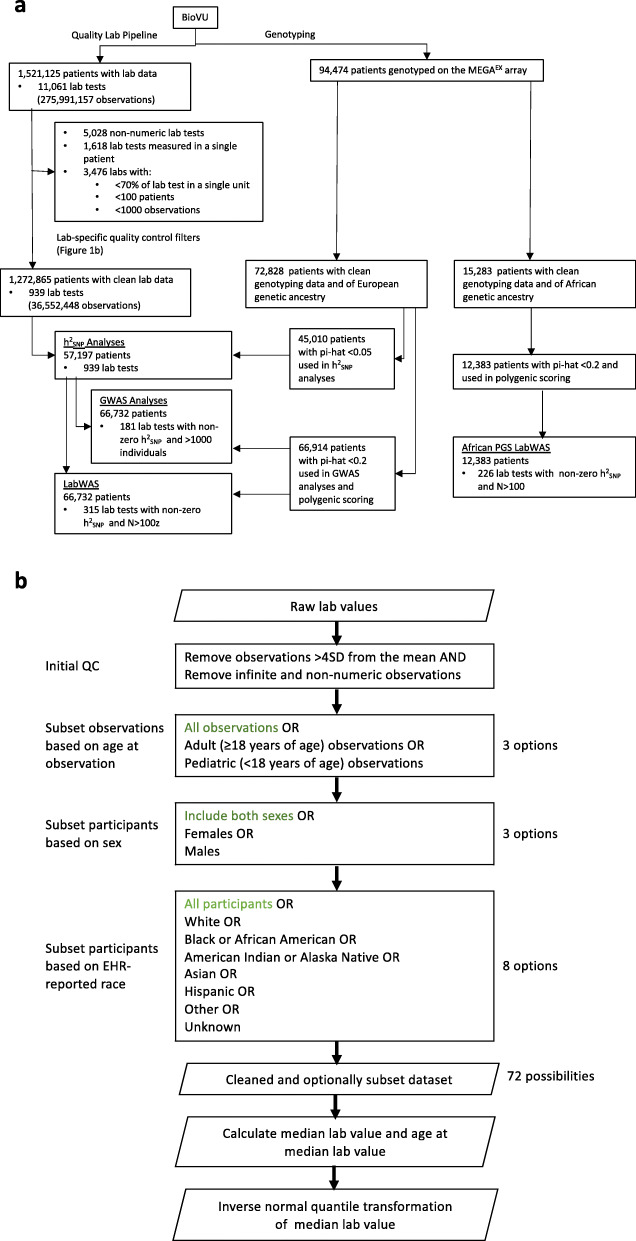
Selection of BioVU patients and datasets for different analyses presented in this manuscript. **a** BioVU patients were selected in parallel for clinical laboratory (lab) test cleaning and for genotyping. **b** Lab-specific quality control filters and subsetting were applied to the 939 lab tests in the 94,474 patients with clean lab data. Parallelograms denote input and output datasets. Options highlighted in green were selected for the proof-of-principle analyses of blood-based lipid lab values

In parallel with the BioVU genotyping project, we extracted data on all lab tests collected in the routine clinical care of 1,521,125 VUMC patients, amounting to 275,991,157 observations across 11,061 lab tests (Fig. 1a). Of these lab tests, 5028 were reported in non-numeric values and 1618 had only been administered to one patient, leaving 4415 quantitative lab tests for further cleaning. Some lab tests had observations recorded in different units (e.g., Selenium reported in both mcg/L and μg/L); thus, we restricted to lab tests for which at least 70% of the observations were measured in the same unit and required that each lab have at least 100 patients and at least 1000 numeric observations, for a total of 939 labs retained for further analysis.

For each of these 939 labs, we applied lab-specific quality control filters (Fig. 1b). First, we filtered infinite and non-numeric values, as well as observations outside of 4 standard deviations from the overall sample mean, indicative of biologically implausible values due to technical or recording errors, monogenic disorders, or extreme environmental influence. We calculated the median lab value for each patient and extracted the patient’s age at median lab value. For patients in whom we had to calculate the median lab value (e.g., those with an even number of observations), we defined the age at median lab value as the mid-point of the patient’s ages at the two lab values used to calculate the median lab value.

The analyses presented in this manuscript use the QualityLab dataset constructed from pediatric and adult observations, in both sexes, in patients of all races (Fig. 1b). In downstream genetic analyses, however, we restrict to participants of European or African genetic ancestry and match the ancestry of the participants in the discovery GWAS used for the training the PGS.

The QualityLab pipeline also provides user with the option to stratify data (Fig. 1b), by age at observation, sex, and EHR-recorded race, for a total of 72 different data subsets. The QualityLab pipeline generates summary statistics and plots for each strata (e.g., mean, maximum, and minimum of the median lab value; Additional file 2: Table S1; Additional file 3: Fig. S1), and returns two versions of the data for downstream analyses. The first is a table of median lab values and age at median lab value for each individual. The second is an inverse normal quantile transformation (INT) of the median lab value data, to account for skewness and non-normality [20, 21]. Importantly, the choice of quality control thresholds is completely in the control of the user. The choices made here reflect the goals of this study which focus on the central tendencies of large populations. However, the outlier thresholds and normalization methods employed here would not be appropriate in a study of rare, potentially pathogenic, variation where large genetic effects and extreme phenotypes may be expected.

### Lab heritability and GWAS analyses

Prior to calculating SNP-based heritability (h^2^_SNP_), we first calculated pairwise relatedness in the BioVU genotyped sample and removed one related individual from pairs with pi-hat greater than 0.05. This stringent threshold was chosen based on prior experience and previously published best practices in the application of restricted maximum likelihood (REML) approaches to the calculation of h^2^_SNP_ [22]. After filtering, 45,010 individuals of European genetic ancestry (Fig. 1a) remained. We then used the genome-wide complex trait analysis (GCTA) package (version 1.92.4) [23] to create a pairwise genetic relationship matrix for all individuals, and heritabilities were calculated using REML methods. We used the median, INT-transformed lab values from the QualityLab pipeline, and of the 481 analyzed labs, 335 demonstrated non-zero heritability. For GWAS analyses, we used a less stringent relatedness filter appropriate to GWAS (pi-hat > 0.2) [24] resulting in a total available sample of 66,732 European descent individuals. Next, we subset to the heritable labs with at least 1000 individuals (*n* = 181) and performed GWAS of the median, INT-transformed lab values using fastGWA [25] (Fig. 1a). All h^2^_SNP_ and GWAS analyses included covariates for sex, cubic splines (knots = 4) of median age across the medical record (to control for non-linear effects of age), and the top 10 principal components of ancestry.

### Heritability and GWAS analyses of lipids

We benchmarked our lipid h^2^_SNP_ estimates against those from two external datasets, the Global Lipids Genetics Consortium (GLGC) [26] and the Million Veterans Program (MVP). GLGC and MVP estimates of h^2^_SNP_ for HDL, LDL, and TG were calculated from GWAS summary statistics using LDSC [27]. We computed h^2^_SNP_ in BioVU using Linkage Disequilibrium Score regression (LDSC) applied to our fastGWA summary statistics for HDL, LDL, and TG (Additional file 3: Fig. S2). However, because LDSC can underestimate h^2^_SNP_ [28], we also calculated h^2^_SNP_ using GCTA. In addition to these h^2^_SNP_ comparisons, we calculated the genetic correlations (*r*_g_) between the BioVU lipid GWASs and the GLGC and MVP lipid GWASs using LDSC and the pre-computed European LD scores from 1000 Genomes Phase 3 European data [29]. We also calculated genetic correlations using a new method, high-definition likelihood [30], which fully accounts for linkage disequilibrium across the genome and is more suitable for traits with lower heritability than LDSC. In sensitivity analyses, we repeated genetic correlations of LDL after controlling the BioVU GWASs for coronary atherosclerosis or diabetes diagnoses, defined as phecodes 411, “Ischemic heart disease,” and 249, “Secondary diabetes mellitus” (Additional file 1).

To validate EHR-based lipid values, we tested the robustness of HDL, LDL, and TG h^2^_SNP_ estimates to different lab value and patient filters. First, we excluded lipid measurements that occurred after the first mention of lipid-altering mediation in the EHR (Additional file 1) and re-calculated each patient’s pre-medication median values of HDL, LDL, and TG. Second, we excluded patients with a diagnosis of CAD, defined by the phecode 411 (Additional file 1).

### LabWAS pipeline

LabWAS uses the median, INT-transformed lab values from the QualityLab pipeline in a linear regression to determine the association with an input variable, adjusting for covariates. In these analyses, a primary goal of the LabWAS was to test common population genetic variation (e.g., PGS) for association with common population variation in lab values. We therefore only included the 335 labs with non-zero h^2^_SNP_. Additionally, we imposed a minimum sample size requirement of 100 for a lab to be included in the LabWAS analysis, bringing the number of labs tested in each scan to 315 in the European ancestry set and 226 in the African ancestry set.

### Polygenic scoring

Prior to polygenic scoring, we randomly removed one related individual from pairs with pi-hat greater than 0.2, leaving 66,732 individuals of European genetic ancestry and 12,383 individuals of African genetic ancestry. (Fig. 1a). We generated lipids PGS for these individuals using PRS-CS [31] with weights derived from the transethnic MVP lipid GWAS summary statistics [4]. PGS for CAD (CAD_PGS_) was calculated using SNP weights from CARDIoGRAMplusC4D GWAS summary statistics [32] using PRS-CS. Because the majority of the MVP transethnic sample was European, linkage disequilibrium was modeled using the pre-calculated European panel. PRS-CS is a recently developed Bayesian polygenic prediction method that imposes continuous shrinkage priors on SNP effect sizes (Polygenic Risk Score – Continuous Shrinkage) [31]. These priors can be represented as global-local scale mixtures of normals which allow the model to flexibly adapt to differing genetic architectures and provide substantial computational advantages. The shrinkage parameter was automatically learnt from the data (i.e., using PRS-CS-auto). SNP effect estimates were obtained from GWAS summary statistics and the score was calculated using a linkage disequilibrium reference panel from 503 European samples in the 1000 Genomes Project phase 3 [19]. Although PRS-CS outperformed other polygenic scoring methods across a range of traits in previous experiments, its superiority may not hold across all genetic architectures [31]. We therefore also generated PGS for the European sample using PRSice-2 [33] (Additional file 1) and have automated a pipeline to generate scores across both methods. PGS were scaled to have a mean of zero and SD of one before testing for association with any outcome variables. We validated each score by testing the proportion of trait variability explained by the PGS, controlling for sex, cubic splines of median age (4 knots) across the medical record, and the top 10 principal components to adjust for genetic ancestry (Additional file 3: Fig. S3).

### LabWAS of polygenic scores

PGS for LDL (PGS_LDL_), HDL (PGS_HDL_), and TG (PGS_TG_) were calculated in BioVU participants using PRS-CS and applying SNP weights from the MVP GWAS summary statistics. We then ran LabWAS of PGS_LDL_, PGS_HDL_, and PGS_TG_ to test whether lipid labs were robustly associated with the genetic scores to which they corresponded. Next, a PGS for CAD (CAD_PGS_) was calculated using SNP weights from CARDIoGRAMplusC4D GWAS summary statistics [32] and a LabWAS of PGS_CAD_ to test whether the score could identify lab traits associated with genetic risk for CAD, before and after controlling for a CAD diagnosis (Additional file 1). Each LabWAS was controlled for sex, cubic splines of median age across the medical record, and the top 10 principal components of ancestry. Results are reported as effect estimates and their 95% confidence intervals per SD increase in the PGS. The Bonferroni-corrected threshold for statistical significance across all tested labs was 3.97× 10^−5^ (0.05/(315 × 4)).

### Replication in Massachusetts General Brigham Biobank

We next sought to replicate the associations between lipids PGS and referent lipids as well as the significant associations with CAD_PGS_ in an external biobank. The MGBB, previously the Partners Biobank, is an ongoing virtual cohort study of patients across the Partners HealthCare hospital system (including Brigham and Women’s Hospital, Massachusetts General Hospital, and other affiliated hospitals), which provides a large-scale resource of linked longitudinal electronic health records (EHR) data, genomic data, and self-reported survey data [34]. All patients provided informed consent before enrollment, and all study procedures were approved by the Partners HealthCare Institutional Review Board.

Lab values were extracted from EHRs and cleaned using QualityLab, resulting in 759 labs for analysis. The median value for each lab trait for each individual was selected and inverse normalized. Lab heritabilities were calculated using REML in GCTA. Of 759 labs that passed QualityLab, 241 demonstrated measurable heritability and included a sample size of at least 100 individuals.

Polygenic scores for HDL, LDL, TG, and CAD were calculated on individuals of European descent in MGBB (*n* = 25,698) using the same criteria as BioVU. Lipids and CAD polygenic scores were associated with each of 234 labs using LabWAS. Lastly, the associations between CAD_PGS_ and lab traits were controlled for CAD diagnosis, defined by phecode 411 (*N* cases = 1094, *N* controls = 20,405). All associations were controlled for sex, top 10 principal components, and the first two splines of median age across the medical record.

## Results

### QualityLab pipeline

A total of 94,474 BioVU patients with clean lab data, of whom 66,732 were also of European genetic ancestry were included in the PGS LabWAS analyses (Fig. 1a). These 66,732 patients had data on 939 labs, containing 30,421,498 observations. The median number of unique lab tests per patient was 44, and the median number of lab observations per patient was 201. Slightly more than half of the BioVU patients in the sample were female (55.6%), and the average median age across the EHR was 52.0 years. These BioVU participants included 10,015 CAD cases and 49,702 CAD controls. In the African ancestry sample, 12,383 patients had data on 925 labs, containing 5,367,062 observations. More than half the patients were female (61.6%) and the average median age was 38.5 years. The median number of unique lab tests per patient was 41, and the median number of lab observations per patient was 150 (Additional file 1; Additional file 2: Table S3). Distributions of lipids levels by genetic ancestry are shown in Additional file 3: Fig. S4.

### Heritability and GWAS analyses

Out of 939 clean lab traits, 335 demonstrated non-zero h^2^_SNP_ and the point estimates ranged from 2 × 10^−6^ to 0.98. (Additional file 2: Table S4, Additional file 3: Fig. S5). As a resource for the community, the GWAS summary statistics for the labs with calculable heritability and a minimum sample size of 1000 individuals (*n* = 181) are available in the GWAS Catalog (Study Number: GCP000091; accession numbers GCST90012603 - GCST90012784; accession numbers are listed in Additional file 2: Table S22).

### Heritability and GWAS analyses of lipids

**Fig. 2:**
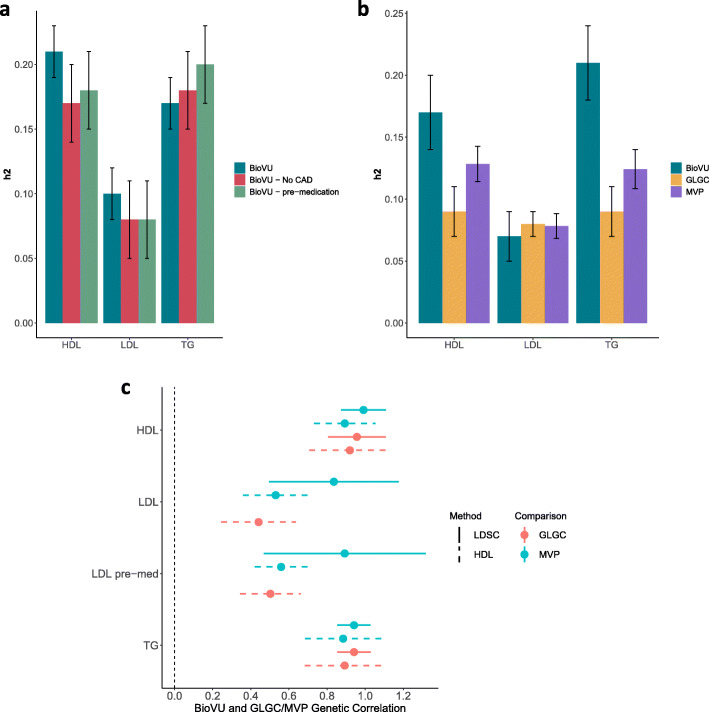
Heritability and GWAS analyses of lipids. **a** Estimates of heritability computed by GCTA in BioVU patients were robust to excluding individuals with a diagnosis of CAD and to removing post-medication observations. **b** Estimates of heritability computed using GWAS summary statistics and LDSC were comparable across BioVU and the Global Lipids Genetic Consortium (GLGC) and Million Veteran’s Project (MVP) samples. **c** Genetic correlations between lipid levels in BioVU and the Global Lipids Genetic Consortium (GLGC) or Million Veteran’s Program (MVP) calculated using LDSC or high-definition likelihood (HDL). Stars denote statistically significant correlations

The h^2^_SNP_ estimates in BioVU were robust to removing post-medication observations, and to removing CAD cases. The number of participants included in these analyses, however, was smaller, and so the standard errors of these h^2^_SNP_ estimates were larger (Fig. 2a; Additional file 2: Table S5). Both GCTA and LDSC gave similar estimates of h^2^_SNP_ in BioVU (Fig. 2b), and the LDSC estimates in BioVU were comparable to those in the GLGC and MVP for all lipids.

Genetic correlation between BioVU and GLGC summary statistics was strong for HDL (LDSC: rg = 0.96, SE = 0.08, *p* value = 2.69 × 10^−35^, high-definition likelihood: rg = 0.92, SE = 0.11, *p* value = 3.25 × 10^−17^) and TG (LDSC: rg = 0.94, SE = 0.05, *p* value = 5.86 × 10^−97^, high-definition likelihood: rg = 0.89, SE = 0.11, *p* value = 7.69 × 10^−17^). When comparing BioVU and MVP, the correlations for HDL (LDSC: rg = 0.99, *p* value = 7.51 × 10^−61^, high-definition likelihood: rg = 0.89, SE = 0.08, *p* value = 2.24 × 10^−27^) and TG (LDSC: rg = 0.94, *p* value = 2.28 × 10^−99^, high-definition likelihood: rg = 0.88, SE = 0.10, *p* value = 4.84 × 10^−18^) were nearly perfect. The LDL and LDL pre-medication genetic correlations between GLGC and BioVU were not calculable using LDSC due to low heritability. Using high-definition likelihood, GLGC LDL levels were significantly correlated when median LDL values across the entire EHR (rg = 0.44, SE = 0.10, *p* value = 1.08 × 10^−5^) and median pre-medication LDL values (rg = 0.50, SE = 0.08, *p* value = 6.38 × 10^−10^). The comparison between BioVU and MVP showed a stronger correlation for LDL (LDSC: rg = 0.84, SE = 0.17, *p* value = 1.47 × 10^−6^; high-definition likelihood: rg = 0.53, SE = 0.09, *p* value = 1.52 × 10^−11^). The genetic correlation with MVP increased when we restricted to pre-medication values of LDL in BioVU (LDSC: rg = 0.89, SE = 0.22, *p* value = 2.90 × 10^−5^; high-definition likelihood: rg = 0.56, SE = 0.07, *p* value = 2.06 × 10^−15^) (Fig. 2c) and increased further when we controlled for coronary atherosclerosis and diabetes diagnoses (GLGC, high-definition likelihood: rg = 0.57, SE = 0.09, *p* value = 8.88 × 10^−9^, MVP, LDSC: rg = 1.00, SE = 0.34, *p* value = 0.004) (MVP, high-definition likelihood: rg = 0.55, SE = 0.09, *p* value = 1.50 × 10^−8^) (Additional file 3: Fig. S6).

### LabWAS of polygenic scores for lipids

**Fig. 3:**
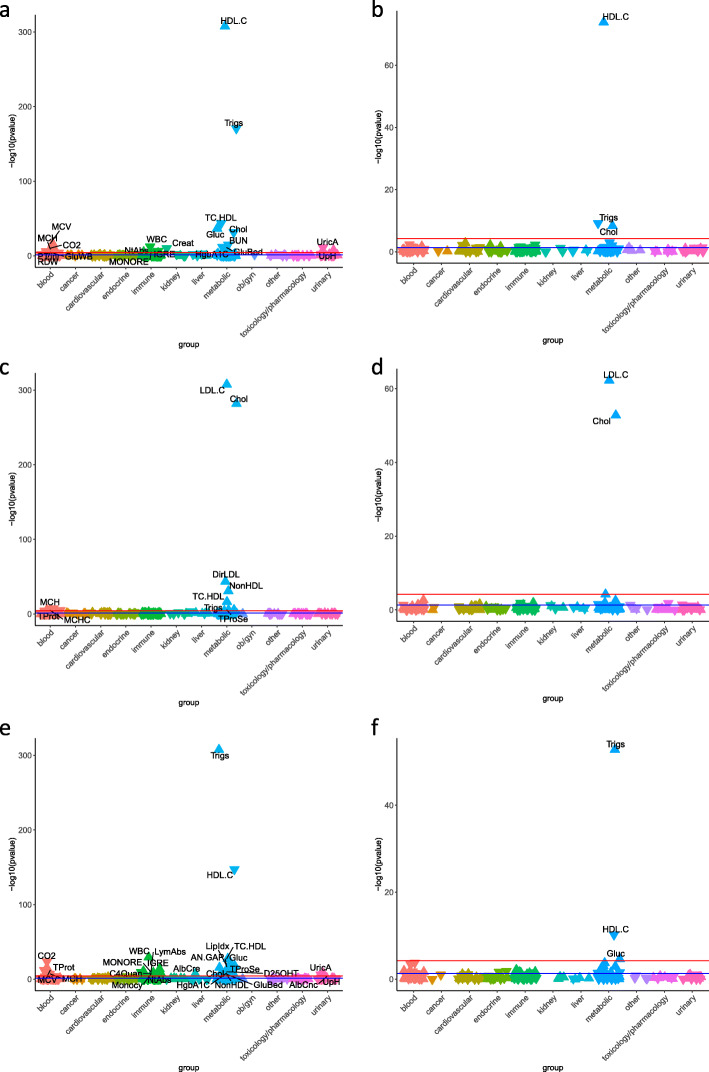
LabWAS of PGS_HDL_ in **a** individuals of European ancestry (EA) and **b** individuals of African ancestry (AA), LabWAS of PGS_LDL_ in **c** EA and **d** AA, and LabWAS of PGS_TG_ in **e** EA and **f** AA. The red line indicates the Bonferroni threshold for statistical significance and the blue line indicates a *p* value of 0.05. Upward triangles indicate that the PGS is associated with increased levels of the lab, while downward triangles indicate an association with reduced levels of the lab

A LabWAS of HDL_PGS_ in the European sample was associated with levels of several metabolic markers (Fig. 3a, Additional file 2: Table S6), including increased HDL (*p* value< 2.23 × 10^−308^, beta = 0.31), decreased TG (*p* value = 2.06 × 10^−171^, beta = − 0.16), decreased total cholesterol to HDL ratio (*p* value = 2.54 × 10^−44^, beta = − 0.22), increased total blood cholesterol (*p* value = 2.51 × 10^−37^, beta = 0.07), and decreased blood glucose (*p* value = 4.62 × 10^−32^, beta = − 0.04), decreased blood urea nitrogen (*p* value = 1.48 × 10^−15^, beta = − 0.03), decreased glycated hemoglobin (*p* value = 1.52 × 10^−12^, beta = − 0.05), decreased bedside glucose (*p* value = 1.03 × 10^−11^, beta = − 0.07), and decreased whole blood glucose (*p* value = 2.49 × 10^−5^, beta = − 0.03). HDL_PGS_ was also associated with four immune labs, white blood cell count (*p* value = 6.14 × 10^−13^, beta = − 0.03), absolute neutrophil count (*p* value = 5.69 × 10^−7^, beta = − 0.03), immature granulocytes (*p* value = 7.86 × 10^−6^, beta = − 0.02), and monocyte to leukocyte ratio (*p* value = 9.13 × 10^−6^, beta = 0.02). Five blood biomarkers associated with HDL_PGS_, mean corpuscular volume (*p* value = 3.48 × 10^−17^, beta = 0.03), blood carbon dioxide (*p* value = 6.69 × 10^−11^, beta = 0.02), mean corpuscular hemoglobin (*p* value = 9.53 × 10^−10^, beta = 0.02), international normalized ratio (*p* value = 1.31 × 10^−6^, beta = − 0.03), and red blood cell distribution width (*p* value = 2.21 × 10^−5^, beta = − 0.02). Finally, three other labs associated with HDL_PGS_, urate (*p* value = 1.13 × 10^−11^, beta = − 0.07), creatinine (*p* value = 1.42 × 10^−10^, beta = − 0.02), and urine pH (*p* value = 2.22 × 10^−8^, beta = 0.02). In the African ancestry group, HDL_PGS_ significantly associated with increased HDL (*p* value = 1.38 × 10^−74^, beta = 0.23), decreased triglycerides (*p* value = 6.72 × 10^−10^, beta = − 0.08), and increased total cholesterol (*p* value = 4.81 × 10^−9^, beta = 0.08) (Fig. 3b, Additional file 2: Table S7).

The LabWAS of LDL_PGS_ showed associations with four lipid labs (Fig. 3c, Additional file 2: Table S8). The most significant association was increased calculated LDL (*p* value < 2.23 × 10^−308^, beta = 0.24), followed by increased total blood cholesterol (*p* value = 1.30 × 10^−282^, beta = 0.20), increased directly measured LDL (*p* value = 3.79 × 10^−44^, beta = 0.19), increased non-HDL cholesterol (*p* value = 1.78 × 10^−31^, beta = 0.19), increased total cholesterol to HDL ratio (*p* value = 5.27 × 10^−17^, beta = 0.13), and increased triglycerides (*p* value = 4.47 × 10^−6^, beta = 0.03). LDL_PGS_ also associated with four blood biomarkers, mean corpuscular hemoglobin (*p* value = 5.68 × 10^−8^, beta = − 0.02), total protein in blood (*p* value = 2.18 × 10^−6^, beta = 0.02), total protein in serum (*p* value = 3.00 × 10^−6^, beta = 0.02), and mean corpuscular hemoglobin concentration (*p* value = 1.50 × 10^−5^, beta = − 0.02). LDL_PGS_ in the African ancestry group associated with LDL cholesterol (*p* value = 5.71 × 10^−63^, beta = 0.24) and increased total cholesterol (*p* value = 1.63 × 10^−53^, beta = 0.21) (Fig. 3d, Additional file 2: Table S9).

The LabWAS of TG_PGS_ was associated with several metabolic measurements (Fig. 3e, Additional file 2: Table S10), including increased TG (*p* value < 2.23 × 10^−308^, beta = 0.28), followed by decreased HDL (*p* value = 4.83 × 10^−148^, beta = − 0.14), increased total cholesterol to HDL ratio (*p* value = 2.95 × 10^−28^, beta = 0.02), increased blood glucose (*p* value = 1.20 × 10^−22^, beta = 0.04), increased lipemic index (*p* value = 1.57 × 10^−18^, beta = 0.01), increased total blood cholesterol (*p* value = 1.25 × 10^−14^, beta = 0.04), increased glycated hemoglobin (*p* value = 5.69 × 10^−9^, beta = 0.04), increased bedside glucose (*p* value = 2.99 × 10^−7^, beta = 0.04), and increased non-HDL cholesterol (*p* value = 1.18 × 10^−6^, beta = 0.08). Additionally, TG_PGS_ showed associations with seven immune labs, white blood cells (*p* value = 3.90 × 10^−30^, beta = 0.04), immature granulocytes (*p* value = 1.99 × 10^−14^, beta = 0.03), absolute lymphocytes (*p* value = 2.01 × 10^−11^, beta = 0.03), monocyte to leukocyte ratio (*p* value = 5.21 × 10^−10^, beta = − 0.03), absolute neutrophils (*p* value = 1.87 × 10^−9^, beta = 0.03), complement C4 (*p* value = 1.03 × 10^−8^, beta = 0.09), and monocyte count (*p* value = 6.76 × 10^−8^, beta = − 0.03). Several blood associations also emerged with TG_PGS_, including carbon dioxide (*p* value = 2.57 × 10^−24^, beta = − 0.04), total protein in blood (*p* value = 4.25 × 10^−16^, beta = 0.03), mean corpuscular volume (*p* value = 9.16 × 10^−13^, beta = − 0.03), mean corpuscular hemoglobin (*p* value = 9.75 × 10^−8^, beta = − 0.02), anion gap (*p* value = 2.03 × 10^−17^, beta = 0.03), total protein in serum (*p* value = 2.61 × 10^–16,^ beta = 0.04), and calcitriol (*p* value = 1.07 × 10^−10^, beta = − 0.05). Lastly, TG_PGS_ associated with albumin to creatinine ratio (*p* value = 9.13 × 10^−8^, beta = 0.10), urate (*p* value = 6.58 × 10^−9^, beta = 0.06), urinary pH (7.66 × 10^−7^, beta = − 0.02), and urinary albumin concentration (*p* value = 2.99 × 10^−5^, beta = 0.06). In the African ancestry group, TG_PGS_ showed significant associations with increased triglycerides (*p* value = 1.66 × 10^−53^, beta = 0.19), decreased HDL cholesterol (*p* value = 6.08 × 10^−11^, beta = − 0.08), and increased glucose (*p* value = 2.33 × 10^−5^, beta = 0.04) (Fig. 3f, Additional file 2: Table S11).

### LabWAS of a polygenic score for coronary artery disease

**Fig. 4:**
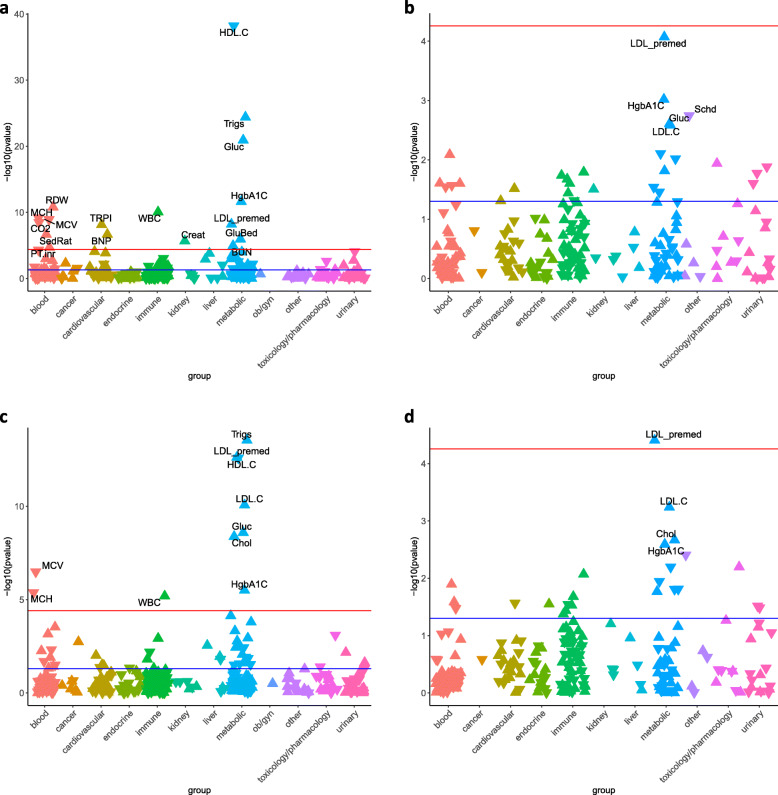
LabWAS of PGS_CAD_ in individuals of **a** European ancestry and **b** individuals of African ancestry. LabWAS of PGS_CAD_ after controlling for CAD diagnosis in individuals of **c** European ancestry and **d** individuals of African ancestry. The red lines indicate the Bonferroni threshold for statistical significance and the blue line indicates a *p* value of 0.05. Upward triangles indicate that the PGS_CAD_ is associated with increased levels of the lab, while downward triangles indicate an association with reduced levels of the lab

We next sought to recapitulate the risk biomarker profile for CAD through a LabWAS of a CAD_PGS_. The CAD_PGS_ reproduced associations, in the direction of risk, with canonical risk factors for CAD (Fig. 4a, Additional file 2: Table S12) in the European ancestry population, including decreased HDL (*p* value = 6.20 × 10^−39^, beta = − 0.07), increased TG (*p* value = 3.98 × 10^−25^, beta = 0.06), increased blood glucose (*p* value = 1.18 × 10^−21^, beta = 0.04) and glycated hemoglobin (*p* value = 2.36 × 10^−12^, beta = 0.05), and bedside glucose (*p* value = 1.10 × 10^−6^, beta = 0.03). The CAD_PGS_ also associated with other known biomarkers of cardiovascular health such as increased troponin-I (*p* value = 7.20 × 10^−9^, beta = 0.04) and brain natriuretic peptide (*p* value = 2.12 × 10^−7^, beta = 0.05). CAD_PGS_ associated with six blood composition markers, red blood cell distribution width (*p* value = 1.60 × 10^−11^, beta = 0.03), mean corpuscular hemoglobin (*p* value = 6.73 × 10^−10^, beta = − 0.02), mean corpuscular volume (*p* value = 1.17 × 10^−9^, beta = − 0.02), carbon dioxide (*p* value = 3.36 × 10^−9^, beta = − 0.02), red blood cell sedimentation rate (*p* value = 2.10 × 10^−7^, beta = 0.05), and international normalized rate (*p* value = 1.96 × 10^−5^, beta = 0.03). Finally, CAD_PGS_ associated with white blood cell count (*p* value = 8.75 × 10^−11^, beta = 0.02), creatinine (*p* value = 2.13 × 10^−6^, beta = 0.02), and blood urea nitrogen (*p* value = 1.09 × 10^−5^, beta = 0.02).

Notably, the CAD_PGS_ was not initially associated with LDL values (*p* value = 0.13, beta = 0.008). The lack of association, however, was attributable to lipid altering medication use and a significant association between the CAD_PGS_ and LDL levels was detected when we restricted to pre-medication values (*p* = 6.19 × 10^−9^, beta = 0.04).

To determine which biomarkers were explained by the clinical presence of CAD as opposed to the genetic risk for CAD, we adjusted the LabWAS of CAD_PGS_ for the coronary atherosclerosis phecode (411) (Fig. 4c, Additional file 2: Table S13). Four canonical biomarkers of CAD risk remained associated with CAD_PGS_ including TG (*p* value = 2.88 × 10^−14^, beta = 0.05), pre-medication LDL (*p* value = 2.40 × 10^−13^ beta = 0.05), HDL (*p* value = 2.55 × 10^−13^, beta = − 0.04), LDL-C (*p* value = 8.48 × 10^−11^, beta = 0.04), blood glucose (*p* value = 2.55 × 10^−9^, beta = 0.02), total cholesterol (*p* value = 4.16 × 10^−9^, beta = 0.03), and glycated hemoglobin (*p* value = 3.16 × 10^−6^, beta = 0.03). The CAD_PGS_ also remained associated with one immune marker, white blood cell count (*p* value = 6.44 × 10^−6^, beta = 0.02), and two other blood biomarkers, mean corpuscular volume (*p* value = 3.23 × 10^−7^, beta = − 0.02) and mean corpuscular hemoglobin (*p* value = 4.18 × 10^−6^, beta = − 0.02).

None of the associations in the initial LabWAS of CAD_PGS_ among African ancestry individuals reached phenome-wide significance; however, three of the top four associations were canonical CAD risk factors including increased glycated hemoglobin A1c (*p* value = 9.56 × 10^−4^, beta = 0.04), increased glucose (*p* value = 0.002, beta = 0.03), and increased LDL cholesterol (*p* value = 0.003, beta = 0.04) (Fig. 4b, Additional file 2: Table S14). When the LDL levels were restricted to pre-medication values, the top association with CAD_PGS_ was pre-medication LDL (*p* value = 8.50 × 10^−5^, beta = 0.06); however, this association did not pass multiple testing correction. After controlling the analysis for CAD diagnosis, the association between CAD_PGS_ and pre-medication LDL surpassed the Bonferroni correction for phenome-wide significance (*p* value = 3.92 × 10^−5^, beta = 0.06) (Fig. 4d, Additional file 2: Table S15).

Lastly, we ran a LabWAS of CAD diagnosis (i.e., using CAD cases/control status (Additional file 1) as the predictor variable) after adjusting for sex and median age across the EHR, which revealed the medical comorbidity pattern of CAD. CAD diagnosis was significantly associated with 136 out of 734 labs in our sample (Additional file 3: Fig. S7, Additional file 2: Table S15), including 34 immune, 32 blood, 24 metabolic, 17 cardiovascular, 8 urinary, 5 toxicology/pharmacology, 4 endocrine, 3 kidney, 3 liver, 1 cancer, and 5 other markers.

### Replication in Mass General Brigham Biobank

In the MGBB, there were 21,499 individuals of European descent with genetic data available with recorded lab data. Slightly more than half of the sample was female (51.5%) and the average age was 56.1 years. The MGBB patients contained 1094 CAD cases and 20,405 CAD controls.

In MGBB, the HDL_PGS_ most strongly associated with HDL cholesterol (*p* value < 2.23 × 10^−308^, beta = 0.33), followed by decreased triglycerides (*p* value = 2.77 × 10^−109^, beta = − 0.17), increased total cholesterol (*p* value = 4.96 × 10^−31^, beta = 0.09), and decreased very low-density lipoprotein (*p* value = 2.62 × 10^−29^, beta = − 0.14). HDL_PGS_ also associated with decreased values of glucose (*p* value = 3.33 × 10^−27^, beta = − 0.07), hemoglobin A1c (*p* value = 3.64 × 10^−18^, beta = − 0.07), and mean glucose value (*p* value = 4.10 × 10^−17^, beta = − 0.07). Additional associations with HDL_PGS_ included cardiac relative risk (*p* value = 5.72 × 10^−17^, beta = − 0.20), alanine aminotransferase (*p* value = 2.45 × 10^−10^, beta = − 0.04), white blood cell count (*p* value = 1.03 × 10^−9^, beta = − 0.04), mean corpuscular volume (*p* value = 6.51 × 10^−8^, beta = 0.03), non-HDL cholesterol (*p* value = 1.41 × 10^−7^, beta = − 0.06), red blood cell distribution width (*p* value = 2.60 × 10^−7^, beta = − 0.03), neutrophils (*p* value = 2.95 × 10^−7^, beta = − 0.03), urate (*p* value = 1.79 × 10^−6^, beta = − 0.05), and alkaline phosphatase (*p* value = 2.01 × 10^−6^_,_ beta = − 0.03) (Additional file 3: Fig. 8a, Additional file 2: Table S17).

The LDL_PGS_ associated with four metabolic labs including LDL-C (*p* value = 1.78 × 10^−158^, beta = 0.24), total cholesterol (*p* value = 2.37 × 10^−158^, beta = 0.20), calculated LDL cholesterol (*p* value = 1.28 × 10^−81^, beta = 0.23), and non-HDL cholesterol (*p* value = 2.90 × 10^−68^, beta = 0.19). The LDL_PGS_ also associated with complement C4 (*p* value = 1.85 × 10^−5^, beta = 0.09), red blood cell sedimentation rate (*p* value = 2.60 × 10^−5^, beta = 0.04), and increased cardiac relative risk (*p* value = 3.80 × 10^−5^, beta = 0.10) (Additional file 3: Fig. 8b, Additional file 2: Table S18).

The TG_PGS_ associated with twelve metabolic labs, including increased measured triglycerides (*p* value < 2.23 × 10^−308^, beta = 0.32), followed by increased very low-density lipoprotein (*p* value = 8.90 × 10^−129^, beta = 0.30), decreased HDL (*p* value = 1.33 × 10^−123^, beta = − 0.17), increased non-HDL cholesterol (*p* value = 8.70 × 10^−28^, beta = 0.12), increased glucose (*p* value = 4.56 × 10^−14^, beta = 0.05), average glucose (*p* value = 4.16 × 10^−10^, beta = 0.05), total cholesterol (*p* value = 1.58 × 10^−9^, beta = 0.05), anion gap (*p* value = 1.52 × 10^−7^, beta = 0.03), total protein (*p* value = 4.63 × 10^−7^, beta = 0.03), globulin in serum (*p* value = 8.80 × 10^−6^, beta = 0.03), aspartate aminotransferase (*p* value = 1.26 × 10^−5^, beta = 0.03), and sodium (*p* value = 1.27 × 10^−5^, beta = − 0.03). TG_PGS_ also associated with seven immune labs, white blood cell count (*p* value = 3.89 × 10^−17^, beta = 0.05), lymphocytes (*p* value = 7.86 × 10^−11^, beta = 0.04), complement C4 (*p* value = 1.58 × 10^−9^, beta = 0.13), automated lymphocyte count (*p* value = 2.14 × 10^−9^, beta = 0.09), neutrophils (*p* value = 3.09 × 10^−7^, beta = 0.05), automated neutrophil count (*p* value = 5.13 × 10^−7^, beta = 0.03), and monocytes (*p* value = 3.38 × 10^−6^, beta = 0.05). Ten additional labs significantly associated with TG_PGS_, including increased cardiac relative risk (*p* value = 5.49 × 10^−15^, beta = 0.19), mean corpuscular volume (*p* value = 3.02 × 10^−14^, beta = − 0.05), glycated hemoglobin A1c (*p* value = 5.00 × 10^−11^, beta = 0.05), urinary pH (*p* value = 9.58 × 10^−10^, beta = − 0.04), red blood cell sedimentation rate (*p* value = 2.22 × 10^−8^, beta = 0.05), alanine aminotransferase (*p* value = 3.88 × 10^−8^, beta = 0.04), alkaline phosphatase (*p* value = 3.17 × 10^−7^, beta = 0.03), blood carbon dioxide (*p* value = 5.63 × 10^−7^, beta = − 0.03), mean corpuscular hemoglobin (*p* value = 1.49 × 10^−6^, beta = − 0.03), and urate (*p* value = 1.62 × 10^−6^, beta = 0.05) (Additional file 3: Fig. 8c, Additional file 2: Table S19).

Finally, the CAD_PGS_ associated with several known CAD risk factors, including decreased HDL-C (*p* value = 1.56 × 10^−21^, beta = − 0.07), increased glucose (*p* value = 9.91 × 10^−15^, beta = 0.05), increased glycated hemoglobin A1c (*p* value = 4.44 × 10^−14^, beta = 0.06), mean glucose (*p* value = 1.75 × 10^−12^, beta = 0.06), and increased triglycerides (*p* value = 2.09 × 10^−12^, beta = 0.05). The CAD_PGS_ also associated with increased red blood cell distribution width (*p* value = 2.42 × 10^−14^, beta = 0.05), increased red blood cell sedimentation rate (*p* value = 4.11 × 10^−9^, beta = 0.05), increased alanine aminotransferase (*p* value = 2.59 × 10^−8^, beta = 0.04), decreased hemoglobin (*p* value = 1.45 × 10^−6^, beta = − 0.03), increased alkaline phosphatase (*p* value = 2.26 × 10^−6^, beta = 0.03), increased white blood cell count (*p* value = 6.77 × 10^−6^, beta = 0.03), decreased albumin (*p* value = 1.06 × 10^−5^, beta = − 0.03), increased globulin (*p* value = 1.29 × 10^−5^, beta = 0.03), decreased iron (*p* value = 3.36 × 10^−5^, beta = − 0.04), and decreased hematocrit (*p* value = 3.77 × 10^−5^, beta = − 0.03) (Additional file 3: Fig. 9a, Additional file 2: Table S20).

After adjusting for CAD diagnosis, CAD_PGS_ remained associated with several heart disease risk factors including decreased HDL-C (*p* value = 8.23 × 10^−16^, beta = − 0.06), increased glucose (*p* value = 6.80 × 10^−11^, beta = 0.04), increased hemoglobin A1c (*p* value = 2.08 × 10^−10^, beta = 0.05), increased mean glucose (*p* value = 2.29 × 10^−9^, beta = 0.05), and increased triglycerides (*p* value = 3.48 × 10^−9^, beta = 0.05). Additionally, associations with red blood cell distribution width (*p* value = 1.40 × 10^−12^, beta = 0.04), alanine aminotransferase (*p* value = 4.01 × 10^−8^, beta = 0.04), red blood cell sedimentation rate (*p* value = 5.50 × 10^−8^, beta = 0.05), alkaline phosphatase (*p* value = 5.44 × 10^−6^, beta = 0.03), serum globulin (*p* value = 4.51 × 10^−5^, beta = 0.03), and white blood cell count (*p* value = 4.67 × 10^−5^, beta = 0.03) remained (Additional file 3: Fig. 9b, Additional file 2: Table S21). In MGBB, the CAD_PGS_ was not associated with levels of LDL-C (*p* value = 0.06, beta = − 0.03), and we were unable to investigate the effects of cholesterol lowering medications on the association.

## Discussion

The results of our study add to a growing body of evidence indicating that lab values from EHRs with linked genetic data can be mined at scale to identify biomarkers for complex disease [1–5]. Our proof-of-principle analyses focused on lipids and CAD in 94,747 genotyped BioVU patients and revealed that EHR lipid values cleaned using our QualityLab pipeline were genetically comparable to those measured in samples ascertained for research. Here, we describe two proof concept studies that demonstrate the power of our proposed discovery paradigm. First, we show that PGS for lipids (HDL, LDL, and triglycerides) associate robustly to their referent lipid across ancestries (Fig. 3). Moreover, the CAD_PGS_ recapitulated associations with known biomarkers in individuals of European ancestry in two biobanks. Unlike CAD, many complex diseases do not yet have bona fide biomarkers, but do have well-powered GWAS that can be used to mine large biobanks and identify quantitative labs which may be correlated, even weakly, with genetic risk for disease. Importantly, the association between CAD_PGS_ and canonical risk factors was significant even among those who did not have a CAD diagnosis. In analyses in Mass General Brigham Biobank, several of the associations with CAD_PGS_ replicated, helping to validate our approach to cleaning and analyzing EHR laboratory data. Interestingly, CAD_PGS_ also associated with white blood cell count, an inflammatory marker that is not currently used to diagnose or monitor heart disease. This association remained after controlling for CAD diagnosis, indicating that CAD genetics could play a role in increasing inflammation. These results highlight the usefulness of our approach which takes advantage of the entire patient population regardless of disease status. This approach offers a potential path forward for the detection of novel biomarkers and for improved understanding of biomarker activity during the prodromal phase of disease. Furthermore, while disease PGS are not diagnostic, they may be useful in identifying pre-symptomatic individuals whose lab values should be monitored more closely.

Furthermore, we show that treatments (in this example, lipid-altering medications) can influence the detection of risk biomarkers at the genetic level. For example, we found that the genetic correlation between LDL measurements in BioVU and MVP increased considerably when we restricted to pre-medication LDL measurements and controlled for CAD or diabetes diagnosis. Additionally, the CAD_PGS_ was strongly associated with pre-medication median LDL values, but was not associated with combined pre- and post-medication median LDL values. This finding also has important and complex implications for the clinical use of PGS recently discussed in the literature [35, 36]. These results indicate that as preventative treatments for complex diseases are adopted (e.g., lipid-altering medications), the risk factors targeted by those treatments (e.g., lipids) are less likely to play a role in the development of subsequent disease (e.g., CAD) in current and future treated populations. Thus, today’s PGS will no longer identify at-risk individuals in future generations who are routinely treated for risk factors which are only now being discovered. Moreover, cases ascertained today for GWAS of diseases with available preventative treatments will be enriched for a different set of genetic (and environmental) risk factors because those individuals with risk factors that can be treated are less likely to develop the disease. PGS, while incredibly valuable, provide only a snapshot of the human genetic profile of complex disease and thus are highly susceptible to these types of cohort effects in addition to other known sources of technical and experimental artifacts [37, 38].

Though the results and approach presented provide an exciting path forward for genetic analysis of EHR-lab data, important limitations should be acknowledged. First, our analyses yielded more associations in patients of European ancestry compared to patients of African ancestry. This is likely to due to decreased power from both the discovery GWASs and the target sample. BioVU has considerably fewer patients of African ancestry than European ancestry, impacting our statistical power to find associations. The polygenic scores of lipids, which were trained on trans-ancestry GWAS summary statistics including individuals of African descent, strongly associated with the referent lipid in the African ancestry sample with effect estimates similar to those found in the European sample. However, the CAD polygenic score, which was trained on a trans-ancestry GWAS that did not include African ancestry samples, yielded far fewer significant associations. These results highlight the critical importance of diversity in GWAS as the downstream applications of such studies are dramatically impacted by representation. As the number of ancestrally diverse GWAS increase, so too will our ability to identify novel biomarkers in different ancestral groups, and the QualityLab pipeline is poised to deliver on these analyses. The QualityLab pipeline could also have more immediate clinical impact for diverse populations. Genetic ancestry, race, sex, age, and ethnicity strongly influence the distribution of lab tests results in healthy people [39], but many current reference ranges were developed using White middle-aged men and are applied to patients irrespective of these differences. This could result in under- or over-diagnosis in some patient groups, and developing lab reference ranges appropriate for diverse demographics is low-hanging fruit for precision medicine. The QualityLab pipeline provides summary metrics based on demographic features which allows the user to evaluate lab distributions across populations, sexes, and ages.

Second, polygenic scores are based on GWAS summary statistics that are typically unadjusted for phenotypic comorbidities. While this approach is optimal in GWAS for many reasons, it introduces the possibility of “phenotypic hitchhiking” in which a comorbid trait is unintentionally selected during the ascertainment of the index trait. Thus, two heritable phenotypes that might share common environmental risk factors but no genetic risk factors can subsequently appear correlated in PGS analysis, even in independent samples. We therefore emphasize that this genetic approach is still fundamentally correlational.

Third, high-throughput analysis of 939 lab traits in our LabWAS required us to prioritize statistical model performance over coefficient interpretability. In our primary analysis, we transformed lab values to fit the normal distribution to improve the performance of the linear regression models [21]. We applied the rank-based inverse normal quantile transformation to all labs, which ensured trait normality by replacing the value of each observation with its quantile from the standard normal distribution. The inverse normal quantile transformation thus preserved the rank ordering of observations, but not the values themselves, and model coefficients therefore are uninterpretable on the original scale. For example, based on our LabWAS results, we are unable to report the change in LDL levels in mg/dL per SD increase in the CAD_PGS_. Multiple testing correction was another statistical challenge inherent to the high-throughput analysis of lab traits. We used the Bonferroni threshold for statistical significance, but this threshold is likely to be overly strict because it ignores the correlation between lab tests.

## Conclusions

Here, we propose that PGS for complex disease can be used to discover genetically related biomarkers of disease by mining quantitative physiological measurements collected during routine clinical testing, but caution that mindful interpretation of correlational results is paramount to progress. We demonstrate the robustness of this discovery paradigm in a proof of principal analysis focused on CAD. As EHR resources grow in size, standardized quality control and analysis pipelines will be necessary to compare results across samples. QualityLab and LabWAS provide a starting point for consistent analysis of lab results stored in various EHR systems. Furthermore, we demonstrated that EHR-derived lipids are similar to measurements ascertained in traditional cohort studies, providing additional rationale for analyses of EHR labs [40]. QualityLab and LabWAS are scalable programs that can be used to confirm clinical paradigms and discover new genetic and environmental relationships between biomarkers and complex traits. We propose that future studies will leverage this discovery paradigm for analysis of rare or understudied complex traits with no known biomarker associations (e.g., psychiatric disorders).

## Supplementary Information

**Additional file 1.** Contains supplementary methods information on genotyping and quality control, definition of coronary artery disease and lipids lowering mediation, and polygenic scoring methods.

**Additional file 2: **Contains all supplementary tables. **Table S1.** Descriptive statistics generated by the QualityLab pipeline. **Table S2.** Lipid-altering medications abstracted from free text in clinical notes. **Table S3.** Characteristics of patients with clean lab and genotyping data. **Table S4.** Estimates of SNP-based heritability and sample sizes for all labs passing QualityLab using REML. **Table S5.** Estimates of SNP-based heritability for blood lipid levels across study populations and estimation methods. **Table S6.** LabWAS of HDL_PGS_ in the European sample in BioVU. **Table S7.** LabWAS of HDL_PGS_ in the African sample in BioVU. **Table S8.** LabWAS of LDL_PGS_ in the European sample in BioVU. **Table S9.** LabWAS of LDL_PGS_ in the African sample in BioVU. **Table S10.** LabWAS of TG_PGS_ in the European sample in BioVU. **Table S11.** LabWAS of TG_PGS_ in the African sample in BioVU. **Table S12.** LabWAS of CAD_PGS_ in the European sample in BioVU. **Table S13.** LabWAS of CAD_PGS_ in the European sample in BioVU controlling for CAD diagnosis. **Table S14.** LabWAS of CAD_PGS_ in the African sample in BioVU. **Table S15.** LabWAS of CAD_PGS_ in the African sample in BioVU controlling for CAD diagnosis. **Table S16.** LabWAS of CAD diagnosis in the European sample in BioVU. **Table S17.** LabWAS of HDL_PGS_ in the European sample in MGB. **Table S18.** LabWAS of LDL_PGS_ in the European sample in MGB. **Table S19.** LabWAS of TG_PGS_ in the European sample in MGB. **Table S20.** LabWAS of CAD_PGS_ in the European sample in MGB. **Table S21.** LabWAS of CAD_PGS_ in the European sample in MGB controlling for CAD diagnosis. **Table S22.** The GWAS Catalog study accession numbers for summary statistics from GWAS of each lab trait.

**Additional file 3: **Contains all supplementary figures. **Figure S1.** QualityLab data visualizations. **Figure S2.** Manhattan and QQ plots from GWAS of BioVU lipids. **Figure S3.**. Predictive abilities of different polygenic scoring methods. **Figure S4.** Lipids levels by genetic ancestry. **Figure S5.** Histogram of BioVU lab heritability estimates. **Figure S6.** LDL genetic correlation sensitivity analyses. **Figure S7.** LabWAS Manhattan of CAD diagnosis. **Figure S8.** LabWAS of Lipids PGS in MGBB. **Figure S9.** LabWAS of CAD PGS in MGBB.

## Data Availability

The data that support the findings of this study are available from Vanderbilt University Medical Center but restrictions apply to the availability of these data, which were used under license for the current study, and so are not publicly available. Data are however available from the authors upon reasonable request and with permission of Vanderbilt University Medical Center. GWAS data generated are available on the GWAS Catalog (accession numbers GCST90012603 - GCST90012784 are listed in Additional file 2: Table S22) [41] and on https://www.dropbox.com/sh/w1pbe0jq1bjkpc5/AAAUIdtBgUybE6iHraE8jvp8a?dl=0. Code for QualityLab and LabWAS software used to generate the results presented in this paper can be found here (https://bitbucket.org/straubp_vandy/quality_labs/) [42] and here (https://bitbucket.org/juliasealock/labwas/) [43]. LabWAS plots can be viewed interactively here: https://dennislab.ca/labwas-in-electronic-health-records/

## Abbreviations

Lab: Laboratory
EHR: Electronic health record
PGS: Polygenic score
LabWAS: Lab-wide association scan
PheWAS: Phenome-wide association scan
VUMC: Vanderbilt University Medical Center
HDL: High-density lipoprotein
LDL: Low-density lipoprotein
TG: Triglycerides
CAD: Coronary artery disease
ICD: International Classification of Disease
CPT: Current Procedural Terminology
HRC: Haplotype Reference Consortium
GWAS: Genome-wide association scan
INT: Inverse normal quantile transformation
h^2^_SNP_: SNP-based heritability
GCTA: Genome-wide complex trait analysis
REML: Restricted maximum likelihood
GLGC: Global Lipids Genetics Consortium
PRS-CS: Polygenic Risk Score – Continuous Shrinkage
