# Supplementary material for: Clinical laboratory test-wide association scan of polygenic scores identifies biomarkers of complex disease
Source: Genome Med. 2021 Jan 13;13:6. doi: 10.1186/s13073-020-00820-8 (PMC7807864; doi:10.1186/s13073-020-00820-8)
Supplement: Supplementary file 1 — Additional file 1. Contains supplementary methods information on genotyping and quality control, definition of coronary artery disease and lipids lowering mediation, and polygenic scoring methods. [file 13073_2020_820_MOESM1_ESM.docx]

## **Supplementary Methods**

## Genotyping and Quality Control

Autosomes were imputed to the HRC panel using Michigan Imputation Server^1^ in five batches. After imputation, genotypes were converted to hard calls with PLINK^2^ using the default threshold settings. SNPs with multiple alleles or imputation quality less than R^2^ of 0.3 were excluded. Next, SNPs with minor allele frequency less than 0.005 or genotyping rates less than 0.98 were excluded. Individuals with call rates less than 0.98 were excluded.

We ran a series of principal component analyses to determine BioVU individuals of European genetic ancestry. First, we performed PCA using FlashPCA2^3^ on BioVU combined with CEU, YRI, and CHB reference sets from 1000 Genomes Project Phase 3^4^. Principal components were scaled so that the axes could be interpreted as proportions of genetic ancestry. To define the European cluster, we selected BioVU individuals who were within 40% of the CEU cluster along the CEU-CHB axis and within 30% of the CEU cluster on the CEU-YRI axis, generating a once-PCA filtered European set. The African cluster was defined as individuals within 70% of the YRI cluster on the CEU-YRI axis and within 50% of the YRI cluster on the YRI-CHB axis.

To ensure subsequent steps would remove SNPs associated with reduced quality rather than cryptic population substructure, we filtered the previously identified BioVU European cluster to identify individuals falling within the CEU, TSI, and GIH 1000 genomes populations, producing a twice-filtered European set. We separately filtered African samples to be greater on -0.1 on PC1 and greater than -0.025 on PC2, producing a twice-filtered African set. Using the twice-filtered sets we conducted a series of SNP checks in the ancestry groups separately. First, we filtered individuals with IBS greater than 0.2 and calculated principal components to use as covariates. Next, we checked for imputation batch effects by conducting pairwise logistic regression of the five imputation batches using sex and top 10 principal components as covariates. SNPs with p-values less than 0.001 in the additive model were flagged. We then compared MAF between BioVU European-CEU reference population and the BioVU African-YRI reference population. Any SNPs with a MAF difference greater than 0.1 were flagged. SNPs with a Hardy-Weinberg Equilibrium p-value less than 10e-10 were flagged. Finally, the flagged SNPs from the batch effect, MAF difference, and HWE were excluded from the once-PCA filtered BioVU sets, resulting in 6,303,629 SNPs on 72,824 individuals of European genetic ancestry and 12,798,111 SNPs on 15,283 individuals of African genetic ancestry.

## Lipid Lowering Medication Use

Lipid-lowering medications were defined by expert consensus (Supplementary Table 2), and medication type and date were abstracted from free text in clinical notes via an in-house natural language processing tool, MedEx^5^. Pre-medication values of HDL, LDL, and TG were those that occurred before the first mention of lipid-altering medications in the EHR.

## Coronary Artery Disease Definition

We defined coronary artery disease (CAD) cases and controls in BioVU participants by the phecode 411, “Ischemic Heart Disease”, which includes unstable angina, angina pectoris, myocardial infarction, and coronary atherosclerosis (https://phewascatalog.org). We mapped ICD-9 and ICD-10 billing codes in BioVU participants to phecodes using the phecode map inherent to the PheWAS v0.99 R package^6^. CAD cases were patients with two or more different ICD codes that mapped to the 411 phecode, or patients with at least two separate occurrences (i.e., on different days) of a single ICD code that mapped to the 411 phecode. This minimum ICD code requirement has been shown to improve the positive predictive value of phecodes^7^. The control group excluded patients with only one component ICD-9 code, or with one or more ICD-9 codes that mapped to related phecodes (as defined by the Phecode Map v1.2).

## Polygenic Scoring Methods

For PRS-CS we used the linkage disequilibrium reference panel from 503 European samples in the 1000 Genomes Project. For PRSice, we used the default r^2^ threshold of 0.1 and tested p-value thresholds of 1, 0.05, 5x10^-4^, and 5x10^-8^.

In our comparison of polygenic scoring methods for lipids, PRS-CS-auto outperformed PRSice at various p-value thresholds. The PGS_LDL_ explained 5.38% of the variance in measured LDL, the PGS_HDL_ explained 9.12% of the variance in HDL, the PGS_TG_ explained 7.86% of the triglycerides measurement, and the PGS_CAD_ explained 2.04% of the CAD diagnosis (Supplementary Figure 3).

# References in Supplementary Material

1 Das, S. *et al.* Next-generation genotype imputation service and methods. *Nat Genet* **48**, 1284-1287, doi:10.1038/ng.3656 (2016).

2 Purcell, S. *et al.* PLINK: a tool set for whole-genome association and population-based linkage analyses. *Am J Hum Genet* **81**, 559-575, doi:10.1086/519795 (2007).

3 Abraham, G., Qiu, Y. & Inouye, M. FlashPCA2: principal component analysis of Biobank-scale genotype datasets. *Bioinformatics* **33**, 2776-2778, doi:10.1093/bioinformatics/btx299 (2017).

4 1000 Genomes Project Consortium *et al.* A global reference for human genetic variation. *Nature* **526**, 68-74, doi:10.1038/nature15393 (2015).

5 Xu, H. *et al.* MedEx: a medication information extraction system for clinical narratives. *J Am Med Inform Assoc* **17**, 19-24, doi:10.1197/jamia.M3378 (2010).

6 Carroll, R. J., Bastarache, L. & Denny, J. C. R PheWAS: data analysis and plotting tools for phenome-wide association studies in the R environment. *Bioinformatics* **30**, 2375-2376, doi:10.1093/bioinformatics/btu197 (2014).

7 Wei, W. Q. *et al.* Combining billing codes, clinical notes, and medications from electronic health records provides superior phenotyping performance. *J Am Med Inform Assoc* **23**, e20-27, doi:10.1093/jamia/ocv130 (2016).

8 Yang, J., Lee, S. H., Goddard, M. E. & Visscher, P. M. GCTA: a tool for genome-wide complex trait analysis. *Am J Hum Genet* **88**, 76-82, doi:10.1016/j.ajhg.2010.11.011 (2011).

9 Willer, C. J. *et al.* Discovery and refinement of loci associated with lipid levels. *Nat Genet* **45**, 1274-1283, doi:10.1038/ng.2797 (2013).

10 Bulik-Sullivan, B. K. *et al.* LD Score regression distinguishes confounding from polygenicity in genome-wide association studies. *Nat Genet* **47**, 291-295, doi:10.1038/ng.3211 (2015).
