# Supplementary material for: Clinical laboratory test-wide association scan of polygenic scores identifies biomarkers of complex disease
Source: Genome Med. 2021 Jan 13;13:6. doi: 10.1186/s13073-020-00820-8 (PMC7807864; doi:10.1186/s13073-020-00820-8)

Fig. S1. Data visualizations are generated by default by the QualityLab pipeline. Pictured are the visualizations for HDL, measured in mg/dL, in 70,639 patients with clean HDL lab values identified by the QualityLab pipeline.

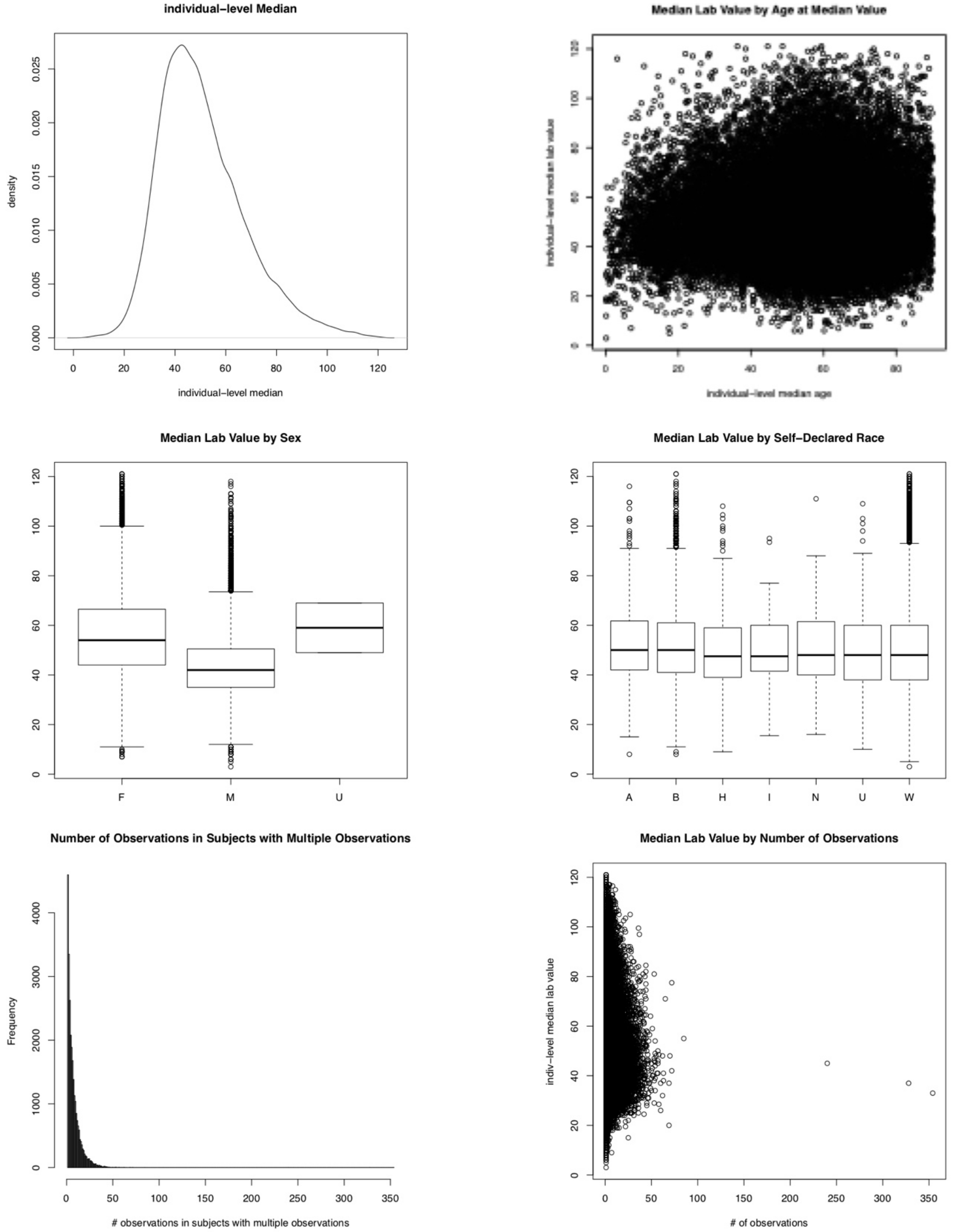

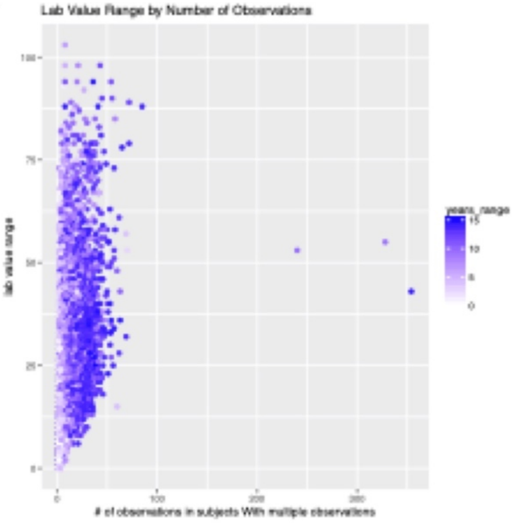
Lab Value Range by Number of Observations
lab value range
# of observations in subjects With multiple observations
years_range

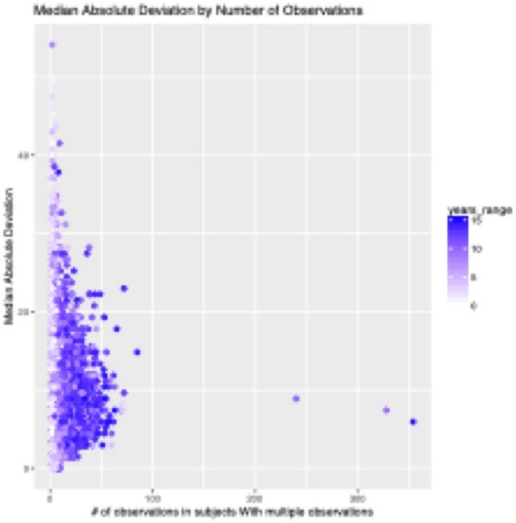
Median Absolute Deviation by Number of Observations
Median Absolute Deviation
# of observations in subjects With multiple observations
years_range

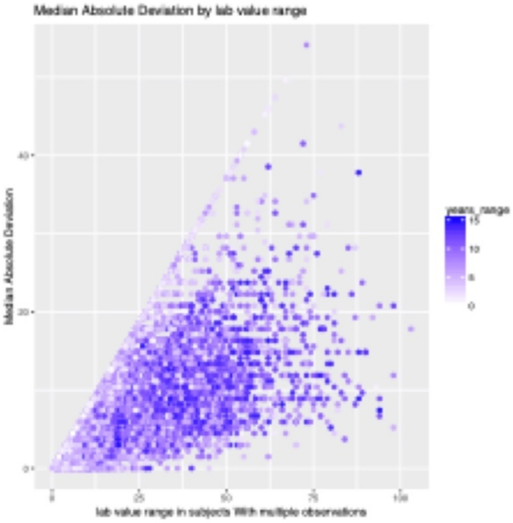
Median Absolute Deviation by lab value range
Median Absolute Deviation
lab value range in subjects With multiple observations
years_range

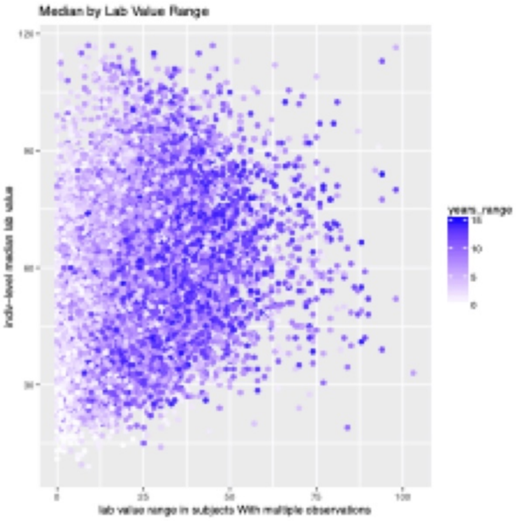
Median by Lab Value Range
indiv-level median lab value
lab value range in subjects With multiple observations
years_range

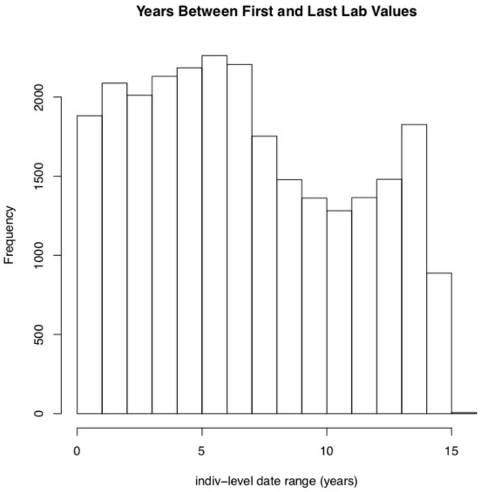
Years Between First and Last Lab Values
Frequency
0
500
1000
1500
2000
0
5
10
15
indiv–level date range (years)

Fig. S2. QQ-plot and Manhattan plots from GWAS of LDL (a), HDL (b), and TG (c) in BioVU. All GWAS were adjusted for sex, cubic splines of median age across the medical record, and the top 10 principal components of ancestry.

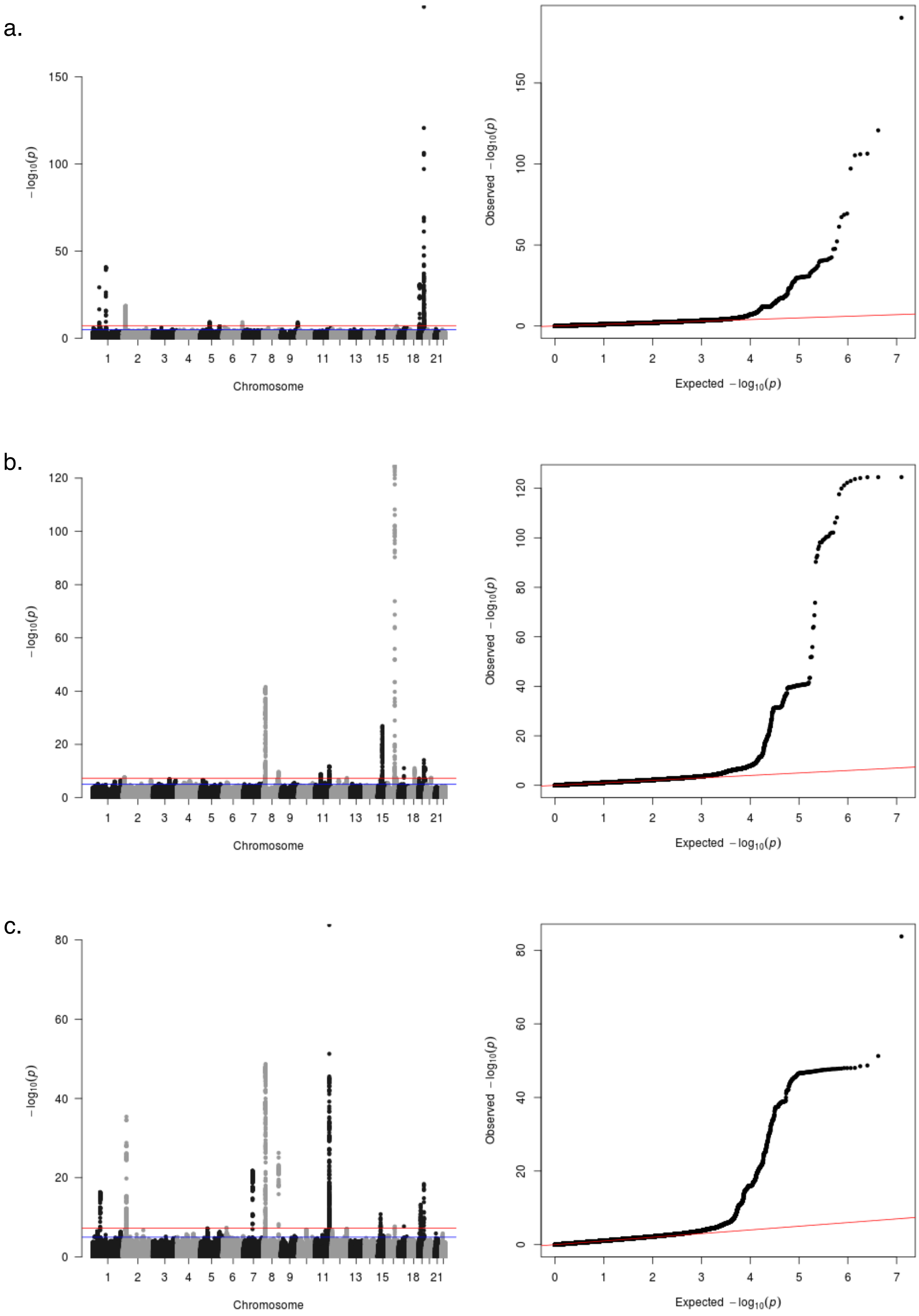

Fig. S3. Predictive abilities of polygenic scores calculated by PRScs and PRSice. Predictive ability is measured as the proportion of same-trait variability explained (R^2) by PGS_{HDL} (a), PGS_{LDL} (b), PGS_{TG} (c), and PGS_{CAD} (d).

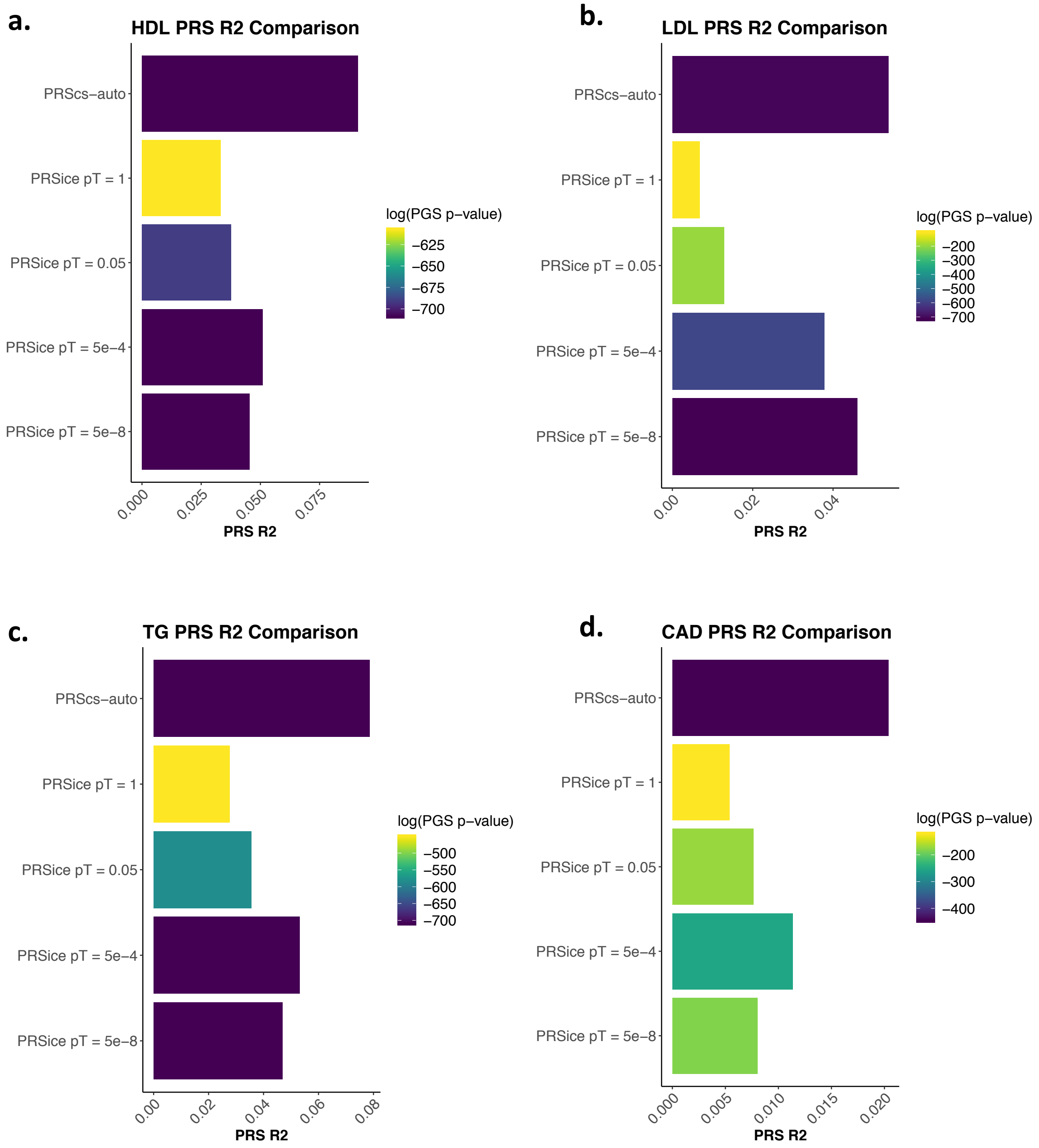

Fig. S4. Levels of a) HDL-C, b) LDL-C), and c) triglycerides stratified by genetic ancestry.

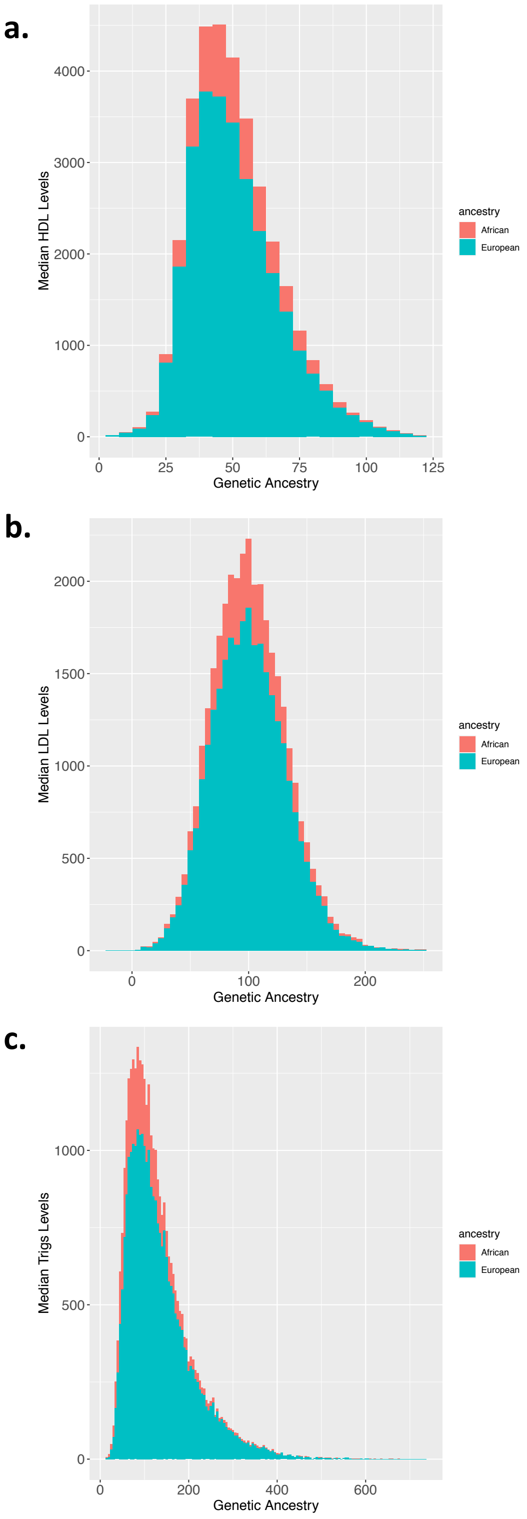

Fig. S5. Histogram of heritability estimates of BioVU labs.

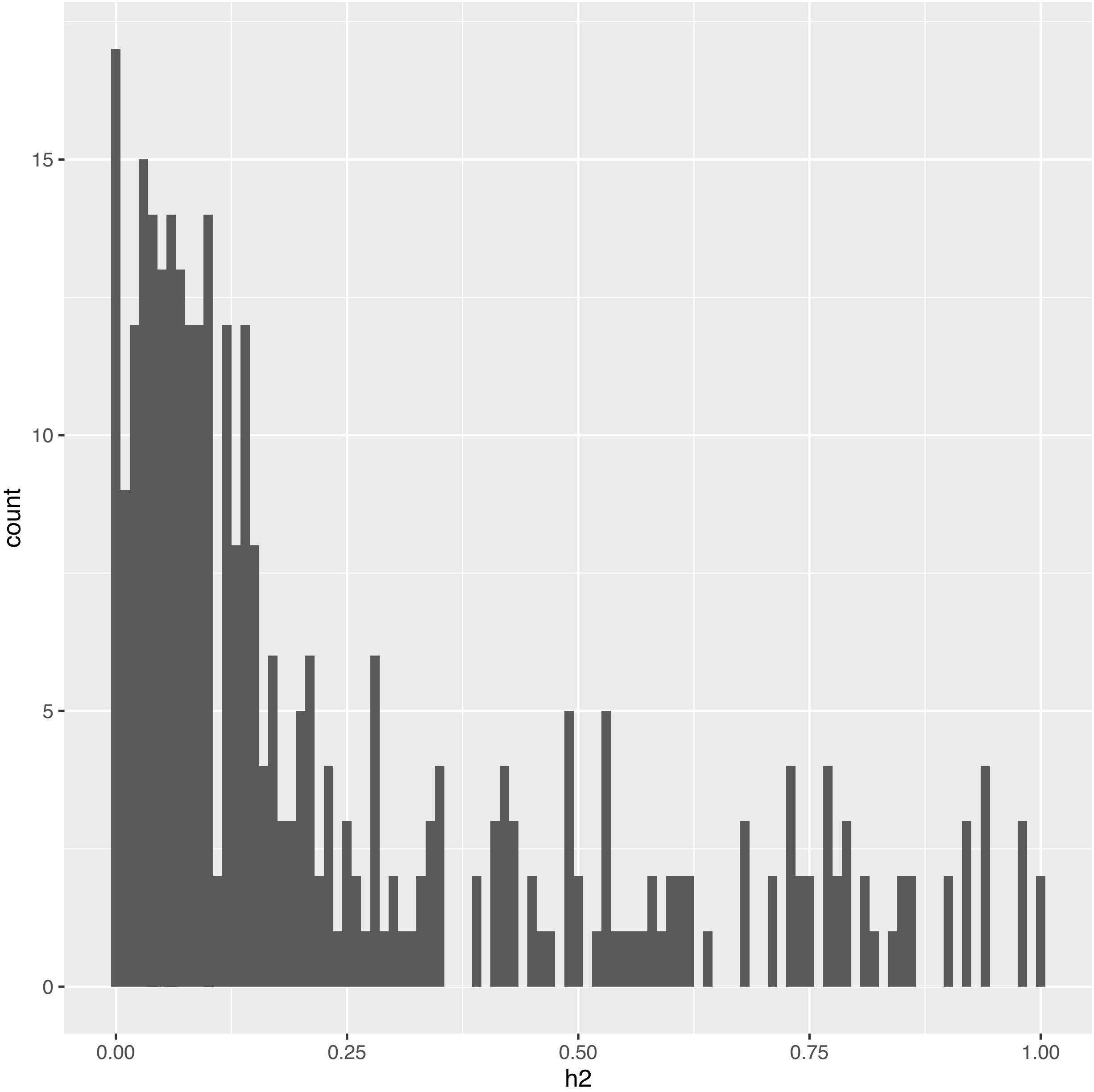

Fig. S6. Genetic correlation between GLGC/MVP LDL and BioVU LDL controlled for CAD and diabetes diagnosis using LDSC (solid) and High-Definition Likelihood (dashed).

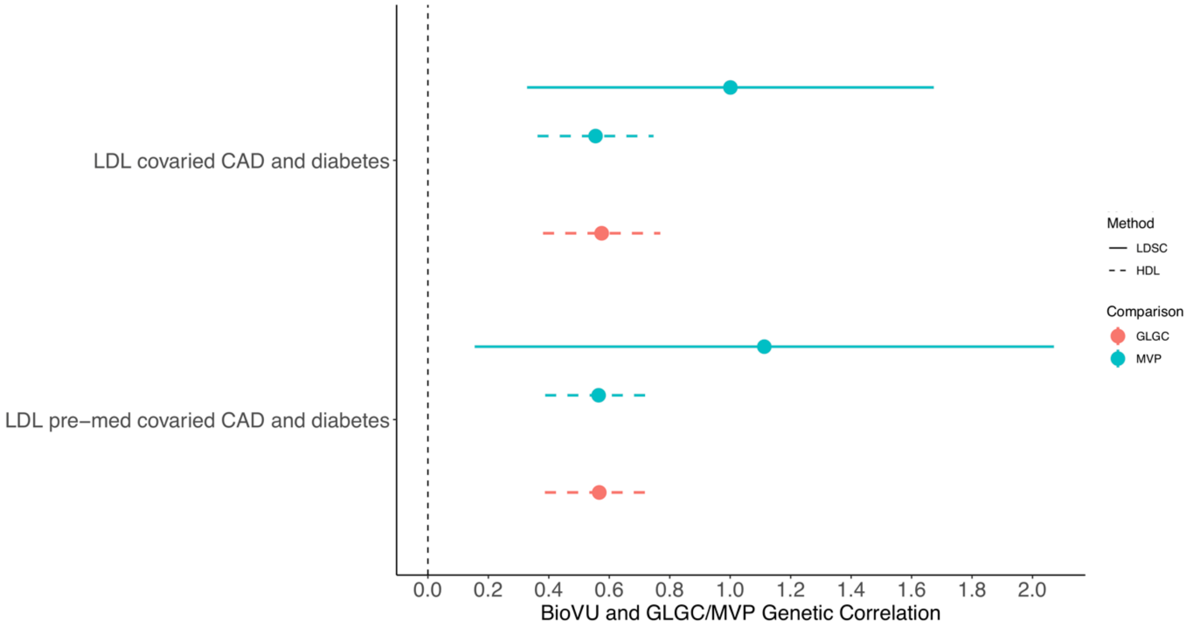

Fig. S7. LabWAS of CAD diagnosis. The red line indicates the Bonferroni threshold for statistical significance (p-value of 6.81x10-5) and labs with p-values below this threshold labelled. The blue line indicates a p-value of 0.05. Upward triangles indicate that a CAD diagnosis is associated with increased levels of the lab, while downward triangles indicate an association with reduced levels of the lab.

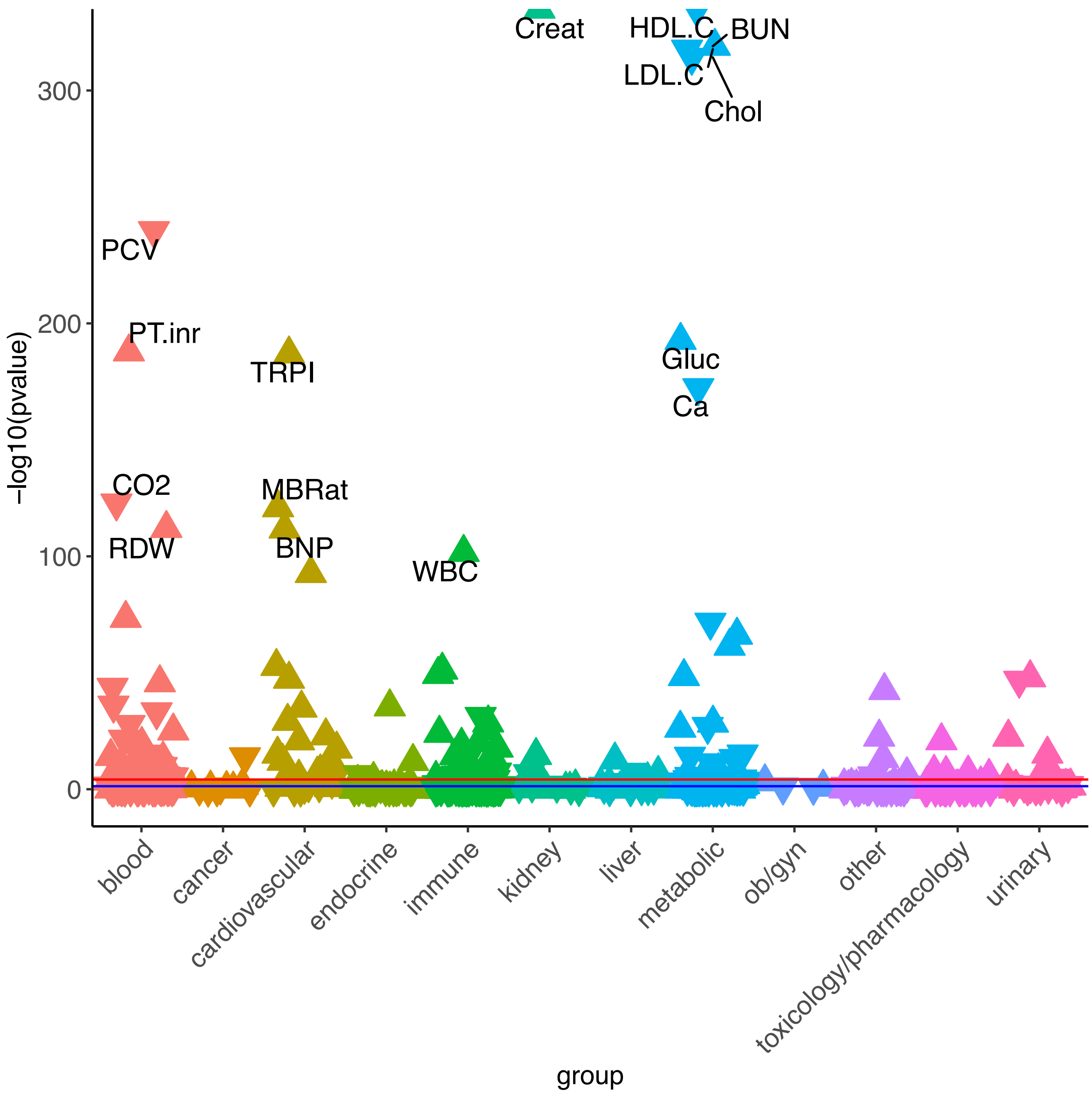

Fig S8. LabWAS of lipids polygenic scores a) HDL, b) LDL, c) TG in Mass General Brigham Biobank. Associations were controlled for sex, top 10 principal components, and cubic splines of median age across the medical record. The red line indicates Bonferroni significance (5.19 x 10^{-5}) and the blue line indicates a p-value of 0.05.

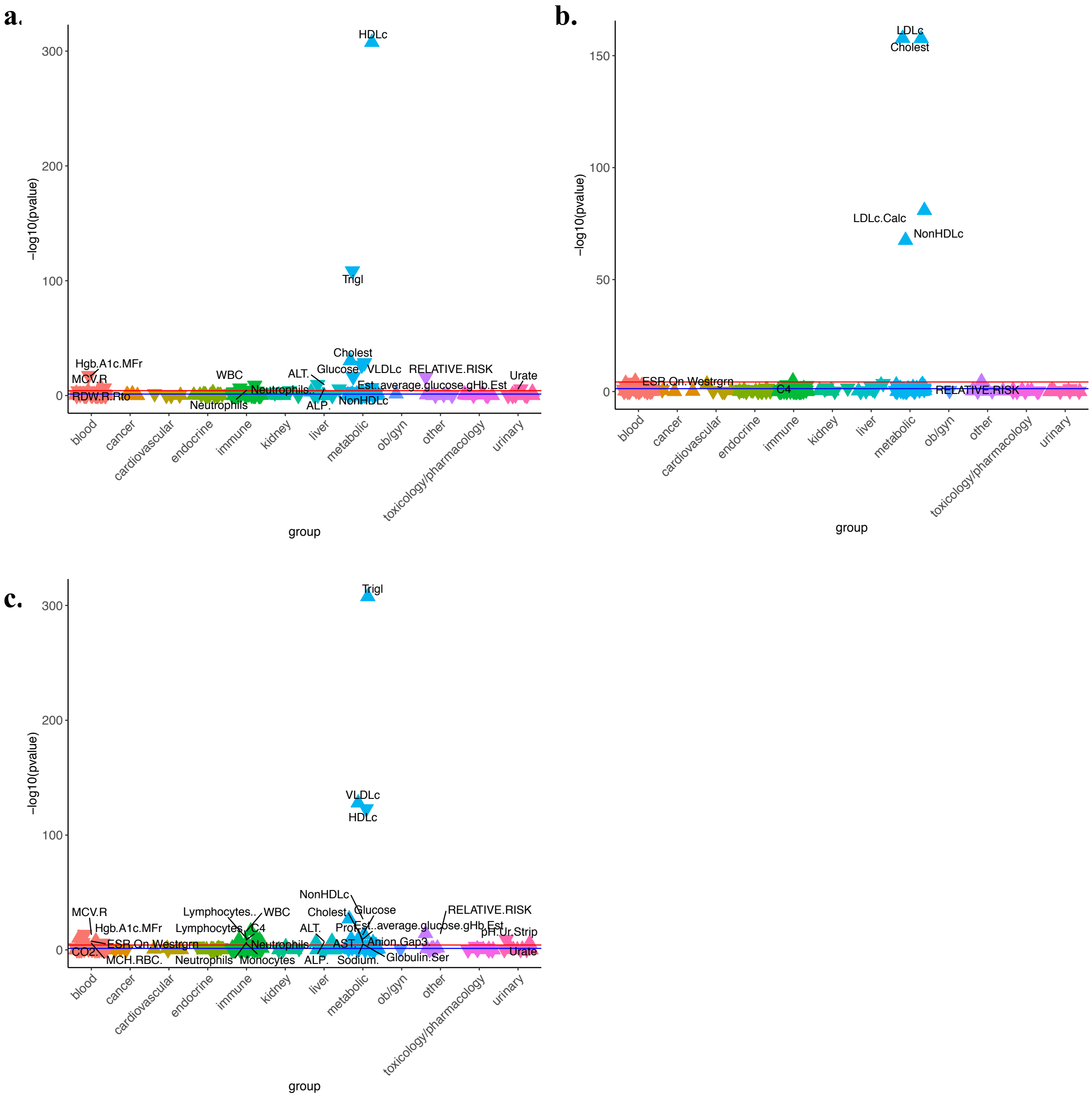

Fig. S9. LabWAS of lipids polygenic scores a) CAD and b) CAD controlled for CAD diagnosis (phecode 411) in the Mass General Brigham Biobank. Associations were controlled for sex, top 10 principal components, and cubic splines of median age across the medical record. The red line indicates Bonferroni significance (5.19×10^{-5}) and the blue line indicates a p-value of 0.05.

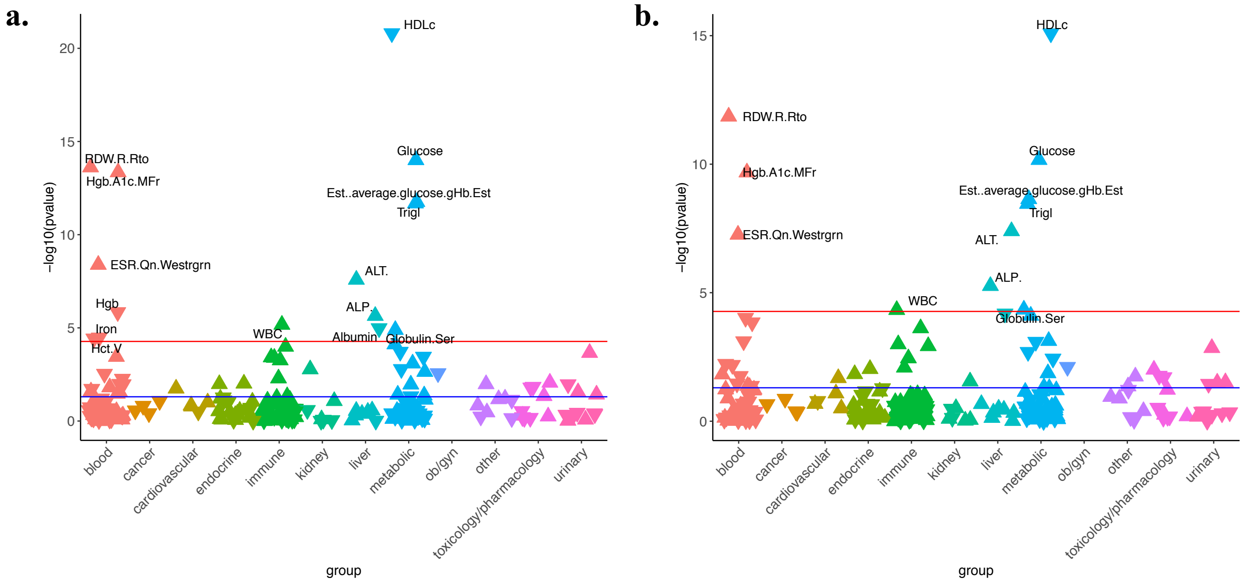

Supplement: Supplementary file 3 — Additional file 3: Contains all supplementary figures. Figure S1. QualityLab data visualizations. Figure S2. Manhattan and QQ plots from GWAS of BioVU lipids. Figure S3.. Predictive abilities of different polygenic scoring methods. Figure S4. Lipids levels by genetic ancestry. Figure S5. Histogram of BioVU lab heritability estimates. Figure S6. LDL genetic correlation sensitivity analyses. Figure S7. LabWAS Manhattan of CAD diagnosis. Figure S8. LabWAS of Lipids PGS in MGBB. Figure S9. LabWAS of CAD PGS in MGBB. [file 13073_2020_820_MOESM3_ESM.pdf]
